# Research funding for newborn health and stillbirths, 2011–20: a systematic analysis of levels and trends

**DOI:** 10.1016/S2214-109X(23)00379-0

**Published:** 2023-10-17

**Authors:** Priyesh Agravat, Eva M Loucaides, Meghan Bruce Kumar, Anna Howells, Alexandra Molina García, Ismail Sebina, Núria Balanza, Elizabeth J A Fitchett, Joy E Lawn

**Affiliations:** a St George's, University of London, London, UK; b London School of Hygiene & Tropical Medicine, London, UK; c KEMRI-Wellcome Trust, Nairobi, Kenya; d King's College Hospital NHS Foundation Trust, London, UK; e Centro de Salud San Fernando, Servicio Madrileño de Salud, Madrid, Spain; f QIMR Berghofer Medical Research Institute, Herston, QLD, Australia; g ISGlobal, Hospital Clínic - Universitat de Barcelona, Barcelona, Spain

## Abstract

**Background:**

Worldwide, an estimated 4·4 million newborn deaths and stillbirths occurred in 2020, and 98% of these deaths occurred in low-income and middle-income countries (LMICs). We aimed to analyse new research grants for newborns and stillbirth awarded by major funders in 2019–20, and all research funding allocated to LMIC-based institutions in 2011–20.

**Methods:**

For this systematic analysis, we searched Dimensions, the world's largest research funding database, for grants relevant to neonatal and stillbirth research. Included grants were categorised by in-depth content analysis, with descriptive quantitative analyses by funder and recipient countries, research pipeline, topic, and year.

**Findings:**

Globally, in 2019–20, major funders awarded a mean annual total of US$577·1 million per year for newborn and stillbirth research (mean total of 550 grants per year). $166·3 million (28·8%) of $577·1 million was directed to small and vulnerable newborn research, but only $8·4 million (1·5%) was directed to stillbirth research. The majority of funding, $537·0 million (93·0%), was allocated to organisations based in high-income countries. Between 2011 and 2020, LMIC-based recipients were named on 1985 grants from all funders worth $486·7 million, of which $73·1 million (15·0%) was allocated to small and vulnerable newborn research and $12·0 million (2·5%) was allocated to stillbirth research. Most LMIC funding supported preclinical or observational studies ($236·8 million [48·7%] of $486·7 million), with implementation research receiving only $13·9 million (2·9%).

**Interpretation:**

Although investment in research related to neonatal health and stillbirths has increased between 2011 and 2020, there are marked disparities in distribution geographically, between major causes of mortality, and among research pipeline types. Stillbirth research received minimal funding in both high-income countries and LMICs, despite a similar number of deaths compared with neonates. Direct investment in LMIC-led research, especially for implementation research, could accelerate the slow global progress on stillbirth prevention and newborn survival.

**Funding:**

None.

**Translations:**

For the French, German and Spanish translations of the abstract see Supplementary Materials section.

## Introduction

In 2020, an estimated 8·6 million children died before 20 years of age, and 4·4 million of these deaths were stillbirths or neonatal deaths combined;^1^ 98% of these deaths occurred in low-income and middle-income countries (LMICs).^2^ Preterm birth, intrapartum complications, and infections are the major causes of neonatal mortality. Moreover, neonatal illness and complications can lead to lifelong health challenges in surviving infants and economic consequences at a societal level.^3^, ^4^, ^5^ Stillbirths are declining at a slower rate than mortality in children younger than 5 years and maternal mortality; an estimated 1·9 million stillbirths occurred in the third trimester in 2020.^2^ Leading risk factors associated with stillbirth are intrapartum complications, maternal infections, and fetal growth restriction.^6^ Although many high-income countries (HICs) and upper-middle-income countries (UMICs) have met WHO's Every Newborn Action Plan target of fewer than 12 stillbirths per 1000 births, most LMICs have not, and many would need a doubling of current progress rates to achieve this goal by 2030.^4^

Despite the sizeable health and economic burden and potential returns, investment to improve newborn health and reduce stillbirths remains inadequate.^7^, ^8^, ^9^ This investment encompasses research funding and other health expenditures (eg, domestic financing, official development assistance [ODA], and out-of-pocket payments), with incomplete data on these different funding sources.^10^, ^11^ ODA funding for reproductive, maternal, newborn, and child health increased from 2003 to 2013 but with persistently low funding directly targeting neonatal health, and stillbirth was mentioned in just nine funding records in 10 years.^7^ Updated analyses have shown similar trends, with aid for reproductive, maternal, newborn and child health declining between 2017 and 2019.^8^

Distinct from programmatic funding, health-related research funding specifically supports new knowledge generation: observational and epidemiological research (eg, defining the burden and describing risk factors); preclinical science and discovery (eg, understanding causes, and innovation in preventive and therapeutic interventions); clinical translation and evaluation of scientific discoveries; optimising implementation methods to deliver these interventions; and improving research infrastructure and capacity.

**Research in context**

**Evidence before this study**

We searched PubMed/MEDLINE with no language or time restrictions using the search terms “(neonatal OR newborn OR baby OR babies OR infant) AND (funding OR funder OR invest OR investment OR finance OR financing OR expenditure OR grant award OR spending OR portfolio)” from database inception to Oct 17, 2022, and found no analyses of research funding for neonatal health and stillbirth prevention. One published study reviewed uptake of WHO global research priorities for newborn health, but without funding amounts. Previous analyses of donor funding, such as the Countdown to 2015 and *The Lancet* Every Newborn Series, have highlighted that although funding for reproductive, maternal, newborn, and child health is increasing, only 13% of donor funding mentioned newborns, with stillbirth terms almost entirely absent. Published analyses predominantly focus on donor financing of implementational programmes, not research funding.

**Added value of this study**

This study is, to the best of our knowledge, the first comprehensive published analysis of research funding worldwide for newborn health and stillbirth, covering both public and philanthropic sources. Dimensions is an inter-linked information system providing the largest global database of research grants and has been used in previous research funding analyses. Through systematic searches and content analysis of grant abstracts in Dimensions, we showed a mismatch in funding allocation to low-income and middle-income countries (LMICs), where 98% of neonatal deaths and stillbirths occur. In 2019–20, the top 13 funders of grants with abstracts mentioning newborn health or stillbirth terms (defined as major funders) awarded a mean annual average of 550 grants, worth US$577·1 million per year globally for research related to newborn health and stillbirths, of which only 29 grants worth $40·1 million per year named LMIC recipients. Between 2011 and 2020, total research funding directly available to LMIC-hosted recipients was just $329·4 million from all funders (ie, an approximate average of $30 million annually). Funding from major funders to all recipients across the research pipeline favoured preclinical, epidemiological, and observational studies, with 20·0% of funding awarded for interventional studies and 4·0% for implementation research. Neonatal infections research received the largest proportion (41·5%) of LMIC-hosted funding in 2011–20, with research related to small and vulnerable newborns receiving 15·0% and stillbirths receiving just 2·5%.

**Implications of all the available evidence**

Considerable funds—more than half a billion dollars annually—are being invested in research related to newborn health. Despite an estimated 1·9 million stillbirths in the third trimester per year, research related to stillbirths (including evaluating implementation methods for known preventive interventions) receives markedly less funding in both high-income countries and LMICs. Funding to LMIC organisations has increased from 2011 to 2020 but this still represents only 7% of annual global funding. Despite the need for evidence-based implementation of effective interventions at scale, implementation research has consistently received a low proportion of total funding. If better allocated, this large amount of research funding presents opportunities to accelerate locally led progress towards newborn survival and stillbirth prevention and meet the UN Sustainable Development Goals by 2030.

Insufficient investment in newborn and stillbirth research has slowed progress in improving survival rates.^4^ Calls for increasing targeted research funding to reduce preventable stillbirths and improve newborn health and survival, especially in LMICs, have been voiced repeatedly.^6^, ^12^ Research priority-setting exercises acknowledge inequities in funding allocation and aim to guide research investment decisions.^13^, ^14^, ^15^, ^16^ However, there are scarce data to guide these decisions, especially on newborn and stillbirth research funding trends according to geography, research topic, pipeline, and disease burden.^17^ Searches of PubMed and MEDLINE with no language or time restrictions found no systematic analyses of global research funding for neonatal health and stillbirth prevention.

We aimed to characterise global research funding for newborn health and stillbirth, describing funding values and the number of grants, focusing on LMICs. Specifically, our objectives were (1) to analyse new global research commitments by major funders for newborn and stillbirth research in 2019–20, and (2) to analyse active research funding for newborns and stillbirths in LMICs in 2011–20.

## Methods

### Data source and search strategy

**Figure 1:**
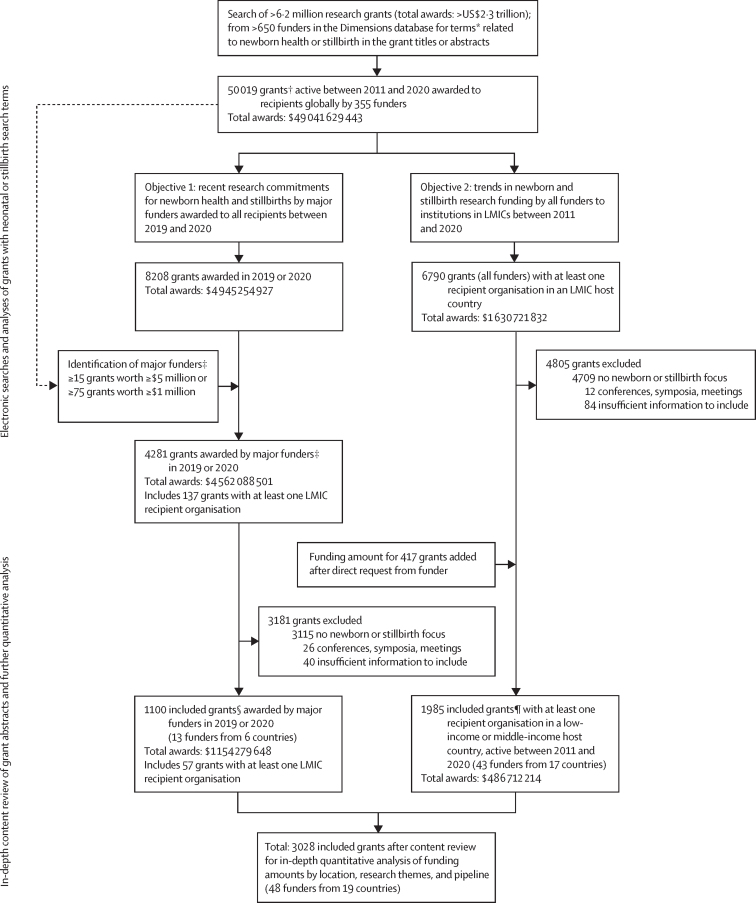
Flowchart of data collection and selection for inclusion in final quantitative analyses *See appendix 4 (pp 2, 19) for newborn and stillbirth search terms. Last search conducted on May 22, 2022. †42 204 grants with available funding amount. ‡Major funders were the Bill & Melinda Gates Foundation, Canadian Institutes of Health Research, the Centers for Disease Control and Prevention (USA), the European & Developing Countries Clinical Trials Partnership, the European Union (European Commission/European Research Council), the National Health and Medical Research Council (Australia), the National Institutes of Health (USA), the National Institute for Health Research (UK), the National Science Foundation (USA), the Research Council of Norway, UK Research and Innovation, US Department of Defense, US Department of Education, and the Wellcome Trust (UK). §1079 grants with available funding amount. ¶1640 grants with available funding amount.

Dimensions is an inter-linked research information system provided by Digital Science containing more than 6·2 million grants (appendix 4 p 2).^18^ For this systematic analysis we conducted an overall search (May 22, 2022) of the Dimensions database for all research grant abstracts related to the neonatal period and stillbirths using key search terms (appendix 4 p 19; figure 1). Search results were restricted to grants active between Jan 1, 2011, and Dec 31, 2020; no language restrictions were applied.

We created two non-mutually exclusive datasets for each objective (figure 1). First, to understand recent funding for newborn health and stillbirth research worldwide, we included all grants awarded in 2019 and 2020 by major funders. Major funders were defined objectively as having awarded either: at least 15 grants worth at least US$5 million each; or, to include smaller-scope but highly active funders, at least 75 grants worth at least $1 million each in records from the overall search.

Second, to analyse more detailed research funding trends for newborn health and stillbirth awarded to LMICs, we selected grants awarded by any funder to at least one LMIC-hosted recipient institution active between Jan 1, 2011, and Dec 31, 2020 (regardless of start or end date).

### Grant screening and categorisation

Grants were screened by title and abstract and further categorised by six reviewers (PA, EML, MBK, AH, AMG, and IS), who were masked to funder and monetary value (figure 1). We included grants describing research (ie, new knowledge generation through investigation of a problem or question) or research-related activities (eg, supporting research infrastructure, workforce development, or stakeholder engagement) related to stillbirth or newborn health (ie, a disease, event, or health-related state occurring or manifesting within 28 days after birth). Grants focusing on maternal outcomes with no stated outcome related to newborn health or stillbirth were excluded, along with grants for symposia or meetings.

The coding methodology was refined during successive rounds of parallel screening and categorisation of randomly selected grants until it reached more than 80% agreement for inclusion. 4531 (41·4%) of 10 934 grants were screened, categorised, and agreed by at least two investigators (of PA, EML, MBK, AH, AMG, or IS), including all high-value grants: those worth at least $2·5 million from major funders in 2019–20 (objective 1; 295 [6·9%] of 4281) and those worth at least $1 million (324 [4·8%] of 6790) for funding to LMIC-hosted recipients in 2011–20 (objective 2). Coders regularly convened to resolve disagreements by consensus.

Missing recipient organisation or funder host countries were manually added by funder and research organisation websites or named researchers’ affiliations as per related contemporaneous publications. 417 missing monetary values for grants in the dataset for objective 2 were addressed through direct information requests to individual funders, reducing missingness for monetary value to 5·1% (345 of 6790 grants).

Included grant abstracts were manually categorised according to thematic area aligned with causes of neonatal mortality and research type (appendix 4 pp 20–26).^19^ The small and vulnerable newborn theme included preterm birth and related complications, growth restriction, and small-for-gestational-age babies.

Host countries of recipient and funder organisations (provided by Dimensions) were categorised according to 2020 World Bank Economy Status and UN Sustainable Development Goal (SDG) Regional Groups.^20^, ^21^

### Data analysis

Data were analysed with Stata (version 17.0). Monetary values were analysed in US$, automatically converted from original currencies with grant start date exchange rates. Grant values were then adjusted for inflation of the US dollar with the annual average Consumer Price Index (US Bureau of Statistics), providing values equivalent to 2020 US$.

For objective 1, the full value of grants from major funders starting in 2019 or 2020 (including commitments beyond 2020) was included to fully capture the direction of research financing by the most influential funders in newborn health.

For objective 2, the total landscape and trends in funding available to LMIC recipient organisations were captured by including any relevant grant active from 2011 to 2020. We assumed grants were dispersed equally throughout their total duration, restricting analyses to funding portions available between 2011 and 2020. Grants with missing monetary value were included in analyses of grant numbers only.

Descriptive analyses of funding were done by research theme, pipeline category, World Bank Economy Status, and UN-SDG region of funder and recipient organisation host countries, including number of grants, median grant size, and total funding value. WHO was considered a HIC funder or recipient unless otherwise specified. When grants were awarded for multiple research themes, pipelines, or organisations, funding was assumed to be evenly distributed. To illustrate country-level funding received for research by burden (annual neonatal deaths and mortality rates), mortality data for 2011–20 were sourced from the UN Inter-Agency Group for Child Mortality Estimation.^22^

### Role of the funding source

There was no funding source for this study.

## Results

Of more than 6·2 million research grants (total value >$2·3 trillion) in the Dimensions database, 50 019 (0·8%) grants were active between 2011 and 2020 and potentially relevant to newborn health or stillbirths (figure 1). 4281 grants were awarded by major funders to global recipients in 2019–20 (objective 1), and 6790 grants, active in 2011–20, had at least one LMIC-hosted recipient organisation (objective 2). Of these 11 071 grants, 7986 were excluded following content analysis, 80 of which appeared in both datasets. 3028 unique research grants awarded by 48 funders were included in this analysis, including 57 grants that appear in both datasets (appendix 4 p 27).

In 2019–20, major funders awarded a mean annual total of 550 new grants related to newborn health or stillbirths to recipients globally, worth a mean annual total of $577·1 million (median award size $473 436; IQR 221 912–1 298 209; appendix 4 p 30).

11 of the 13 major funding organisations that committed this $1·2 billion in 2019 and 2020 were based in the USA or Europe: the top five funders (and the total funding they each awarded in 2019–20) were the US National Institutes of Health ($613·0 million [53·1%]), the Bill & Melinda Gates Foundation ($162·5 million [14·1%]), the European Commission/European Research Council ($84·9 million [7·4%]), the European & Developing Countries Clinical Trials Partnership ($71·9 million [6·2%]), and the Australian National Health and Medical Research Council ($51·0 million [4·4%]; table).

**Table:** Total value of new grants awarded in 2019 and 2020 by the major funders listed

		**Observational clinical research, epidemiology**	**Basic science, preclinical research, technology development**	**Interventional or experimental research**	**Implementation research, complex evaluation, health-systems research**	**Research-related activity, workforce and infrastructure development, stakeholder engagement**	**Unspecified research type**	**Total**
Funder or funder group								
	National Institutes of Health (USA)	281 349	257 208	31 748	15 843	24 540	2355	613 043
	Bill & Melinda Gates Foundation (USA)	24 493	9066	450	10 997	15 178	102 360	162 543
	European Union (European Commission/European Research Council)	38 134	20 511	2416	2407	19 473	1912	84 852
	European & Developing Countries Clinical Trials Partnership	19 916	91	41 102	2543	8282	0	71 934
	National Health and Medical Research Council (Australia)	15 610	10 738	5527	3696	1848	13 613	51 032
	UK Research and Innovation	18 996	11 617	11 851	1985	1532	1320	47 302
	National Institute for Health Research (UK)	6002	359	27 658	7783	2756	0	44 558
	Canadian Institutes of Health Research	13 973	11 711	814	946	1711	631	29 786
	US Department of Defense	5420	16 166	0	0	0	0	21 587
	Wellcome Trust (UK)	9677	4089	3468	218	183	0	17 635
	The Research Council of Norway	897	3796	214	0	216	0	5124
	National Science Foundation (USA)	844	2516	128	0	988	0	4475
	Centers for Disease Control and Prevention (USA)	344	0	0	65	0	0	409
Overall research theme								
	Any grants related to newborn health	432 316	345 567	125 375	46 482	76 625	122 190	1 148 556
	Grants specifically related to newborn period only	82 643	190 786	21 596	4776	10 643	46 665	357 109
	Any grants related to small and vulnerable and newborns	94 816	141 346	25 897	3133	20 769	46 673	332 633
	Any grants related to and including stillbirth theme	9914	2357	4947	887	82	9859	28 046
	Funding specifically allocated to stillbirth theme	6627	2330	2473	444	82	4930	16 885
Specific newborn health research theme								
	Preterm direct complications	71 271	118 112	15 387	1291	16 655	40 133	262 848
	Neonatal infections	107 866	51 645	48 222	5505	7444	10 109	230 791
	Non-specific exposures and outcomes	47 745	7778	18 711	26 644	29 963	21 959	152 800
	Congenital conditions	24 459	76 194	5786	1476	2885	140	110 940
	Other specific exposures and outcomes	56 517	15 230	2757	103	7877	9889	92 372
	Neurological conditions and neurodevelopment	47 667	11 974	3482	250	2732	917	67 022
	Feeding and nutrition	19 077	3742	11 695	2811	4740	21 059	63 124
	Other subspecialty conditions and physiology	18 841	12 544	2770	4396	3488	2215	44 256
	Growth restriction or small for gestational age	15 087	14 198	7377	1471	837	835	39 806
	Intrapartum or birth complications	6831	19 235	3071	2092	0	5862	37 090
	Immunology and the microbiome	11 547	11 993	3530	0	0	2218	29 288
	Neonatal jaundice	2122	2895	113	0	2	1925	7058
Funding received by Sustainable Development Goals super region								
	Europe and northern America	400 507	331 327	95 536	30 672	65 017	94 916	1 017 974
	Sub-saharan Africa	15 479	1316	19 258	12 115	9079	8851	66 098
	Oceania	16 616	12 304	7078	3696	1847	13 613	55 155
	Central and southern Asia	2350	2659	3080	0	0	4810	12 899
	Northern Africa and western Asia	705	0	0	0	760	0	1465
	Eastern and southeastern Asia	0	0	424	0	0	0	424
	Latin America and the Caribbean	0	263	0	0	2	0	266
Funding received by recipient organisation host country World Bank classification								
	Low-income economies	6770	0	14 256	445	4472	128	26 071
	Lower-middle-income economies	3954	3388	6131	11 188	3950	13 447	42 058
	Upper-middle-income economies	7369	850	2374	481	980	86	12 141
	High-income economies	417 563	343 631	102 614	34 368	67 305	108 529	1 074 010
	All funding awarded low-income and middle-income country recipients	18 093	4238	22 761	12 115	9401	13 661	80 269
Total		435 656	347 869	125 375	46 482	76 706	122 190	1 154 280

Data are presented as US$ (thousands). Awards are assumed to be evenly distributed across categories and recipients, where grants covered multiple categories or were awarded to more than one organisation.

**Figure 2:**
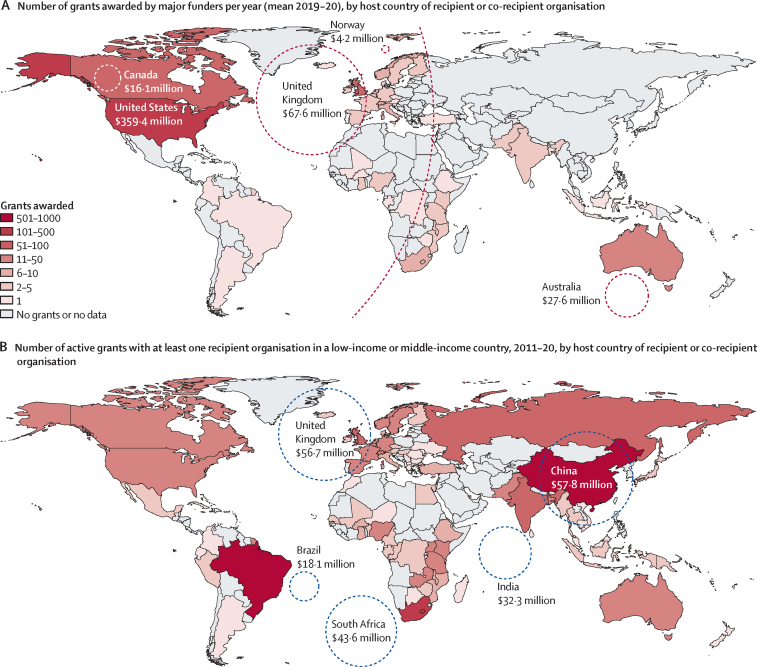
Grants received by country and total funding amounts received by the five most frequent recipient or co-recipient countries Bubble labels show funding awarded (in US$) to the top five recipients according to number of grants, with bubble size proportional to funding received. Funding amounts represent new commitments by major funders per year (mean total 2019–20) in panel A and total active funding from all funders to grants with at least one LMIC recipient in 2011–20 in panel B (any funding from all ongoing grants, restricted to 2011–20). LMIC=low-income and middle-income country.

Total mean annual new funding commitments from major funders in 2019–20 were predominantly to research organisations in the USA ($359·4 million [62·3%] of $577·1 million) and the UK ($67·6 million, 11·7%) both in terms of total value and number of grants (figure 2A). An average of 29 (5·2% of 550) annual grants from major funders ($40·1 million; 7·0%) had one or more LMIC recipients (compared with 530 [96·4%] naming HICs). This funding to LMICs represented 54·5% of the total $74·3 million awarded per year to grants with named LMICs (mean, 2019–20) by all funders (appendix 4 p 37).

In 2019–20, mean annual awards by major funders were $217·8 million (37·7% of $577·1 million) for epidemiological and observational research, $173·9 million (30·1%) for basic science, preclinical research and technology development, $62·7 million (10·9%) for interventional or experimental research, and $23·2 million (4·0%) for implementation research (appendix 4 p 30).

Research themes receiving the highest proportion of mean annual funding from major funders were direct complications of preterm birth ($131·4 million [22·8%] of $577·1 million), neonatal infections ($115·4 million [20·0%]) and non-specific exposures ($76·4 million [13·2%]; appendix 4 pp 30, 44). Research focused on stillbirths received only $8·4 million per year (1·5%) and was evenly distributed across research pipeline categories and recipient location, between Oceania ($3·0 million, 35·2%), Europe and North America ($2·8 million, 33·5%), and sub-Saharan Africa ($2·5 million, 29·2%; appendix 4 pp 30, 35). Research related to small and vulnerable newborns received $166·3 million per year (28·8%; appendix 4 p 30), of which $70·7 million (42·5%) supported basic science, technology development and preclinical research, and $12·9 million (7·8%) supported interventional studies and implementation research (table).

Over the 2011–20 period, 1985 active grants worth $486·7 million (median available funding per grant $37 433; IQR 23 238–87 241) had at least one named LMIC-hosted recipient (appendix 4 p 33). The total annual active funding for research related to newborn health or stillbirths almost tripled, from $23·5 million in 2011 to $68·2 million in 2020 (appendix 4 pp 36, 44). Annual active funding for research related to small and vulnerable newborns increased from $2·7 million (11·6%) of $23·5 million in 2011 to $10·5 million (15·4%) of $68·2 million in 2020; for stillbirth research it increased to $2·8 million (4·1%) of $23·5 million from $0·6 million (2·7%) of $68·2 million over the same period (appendix 4 p 36).

For comparison with major funders’ annual commitments globally in 2019–20, mean new funding from all funders to at least one LMIC recipient (2019–20) was $74·3 million per year (267 mean total grants; appendix 4 p 37).

**Figure 3:**
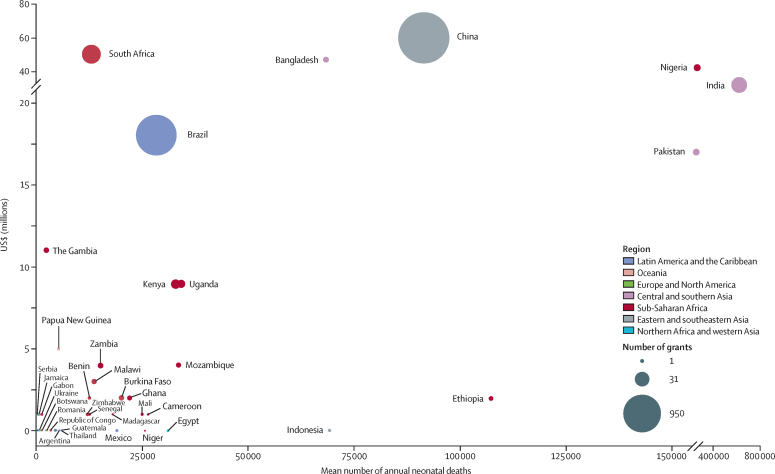
Bubble chart demonstrating the country-level relationship between neonatal mortality and total research investments received by LMIC institutions 2011–20 Mean neonatal mortality rate (2011–20) as defined by neonatal deaths per 1000 live births according to UN IGME data and total research investments received by LMIC institutions 2011–20 (funding amount and number of grants plotted are those specific to LMIC institutions and any funding apportioned to grant partners in HIC is not included here), with bubble size representative of number of grants received. LMIC=low-income and middle-income countries. HIC=high-income countries.

Funding flows identified among grants with at least one LMIC recipient based on World Bank economy groups of funder and recipient organisation host countries are shown in figure 3. 83·4% of funding ($406·1 million of $486·7 million) from 2011 to 2020 was awarded by HIC-based organisations, 14·2% ($69·2 million) by UMICs, and 2·3% ($11·4 million) by lower-middle-income countries. Of all grants with at least one LMIC recipient, only 67·7% of funding ($329·4 million) was directly available to LMIC recipients; 32·3% ($157·3 million) was allocated to recipient organisations in HICs and 26·3% ($127·9 million) was allocated to recipient organisations in UMICs. Upper-middle-income economies funded $68·8 million to others in the same economic group (20·9% of all $329·4 million funding directly available to LMICs), which is more than the $59·1 million awarded to UMICs by HICs. The median funding available per grant to LMIC recipients was $36 670 (IQR 22 763–859 999), whereas the median funding per grant available for HIC co-recipients (named on 108 [5·4%] of 1985 grants) was at least 18 times higher, at $661 511 (IQR 178 613–1 618 847; appendix 4 p 40).

Top funders of LMIC-received grants were the Bill & Melinda Gates Foundation ($117·1 million), the European Union (European Commission/European Research Council; $97·5 million) and UK Research and Innovation ($93·0 million), together accounting for 63·2% ($307·6 million) of total active funding ($486·7 million) from 2011 to 2020 (appendix 4 p 40). China (934 [47·1%] of 1985 grants) and Brazil (584 [29·4%] grants) comprised the top two funder host countries by the number of active grants from 2011 to 2020 (figure 2B), but awards from these sources were smallest in size (median available funding $35 841 from China and $9263 from Brazil; appendix 4 p 40). From grants with at least one LMIC recipient, organisations in sub-Saharan Africa and Asia received $299·1 million (61·5% of $486·7 million) over this time period, with European and North American recipient partners receiving $155·1 million (31·9%) of the total funding (appendix 4 p 33).

From active grants with at least one LMIC recipient between 2011 and 2020, the majority of funding ($236·8 million [48·7%]) supported preclinical ($78·8 million) or observational studies ($158·0 million), with interventional research receiving $99·3 million (20·7%), and implementation research receiving only $13·8 million (2·9%; appendix 4 p 33). We identified 36 (1·8%) of 1985 grants supporting broader research-related and capacity strengthening activities over the decade, collectively worth $49·8 million.

Top research themes mirrored recent commitments by major funders in 2019–20 to all recipients and included neonatal infections ($202·0 million [41·5%] of $486·7 million), non-specific exposures ($117·0 million [24·0%]), and direct complications of preterm birth ($44·4 million [9·1%]; appendix 4 p 33). Investment in neonatal infections research comprised 47·9% of total active funding ($32·7 million of $68·2 million) by 2020, having increased from 28·8% ($6·8 million of $23·5 million) in 2011 (appendix 4 p 36). Stillbirth was among the least funded research themes, receiving $12·0 million (2·5%) over the 10-year period (appendix 4 p 33).

**Figure 4:**
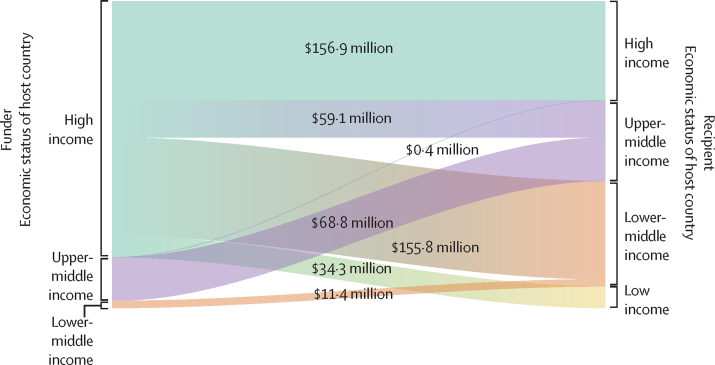
Funding flows for all grants with at least one recipient in an LMIC country, active 2011–20 Funding was allocated to World Bank economy groups according to host country of recipient organisations. Where grants were awarded to multiple organisations, funding was assumed to be evenly distributed between recipients. All funding is in US$ (millions) adjusted for inflation to equate to the US$ 2020 value. Median award size of grants from HIC when partnered with at least one LMIC is $660 000. Median award size of grants received by LMIC-only institutions is $36 000. LMIC=low-income and middle-income countries. HIC=high-income countries.

Brazil, South Africa, and China received more funding than other nations with a similar mean annual number of neonatal deaths (figure 4) or neonatal mortality rates (appendix 4 p 43), mostly from in-country funders. Many sub-Saharan African nations with more than 20 000 annual neonatal deaths received less than $5 million in 2011–20 (figure 4).

## Discussion

To the best of our knowledge, this is the first comprehensive analysis of research funding worldwide for newborn health and stillbirth. These analyses of both public and philanthropic sources show considerable allocated funding; major funders awarded $577·1 million per year (550 grants) in 2019 and 2020 for newborn and stillbirth research, with 93·0% allocated to HIC recipients. In those 2 years, these funders directly allocated only $40·1 million (7·0%) annually to LMIC organisations, which were awarded $74·3 million annually overall. We focused more detailed analyses on trends in research funding available to LMICs between 2011 and 2020. Encouragingly, the annual active funding to LMICs almost tripled, totalling $329·4 million over the 10-year period. Research funding related to stillbirths was persistently low in all settings despite its high-impact potential.^6^

Clear inequities exist in the geographical distribution of research funding from major funders for newborn health and stillbirths; LMIC organisations received an average of 29 (5·2%) of 550 awards annually, compared to an average of 530 (96·4%) of 550 awards to HIC recipients. However, funding flows and relationships by country economic status are neither one-directional, nor linear. UMIC funders provided 20·9% of funding available for LMICs (most commonly to in-country recipients), especially in BRICS nations—the most research-active LMICs by grant number. Our analyses describe the recipients’ location, not the location of the research; however, the high proportion (32·3%) of available funding naming LMIC recipients received in HICs suggests HIC funders’ tendency to rely on in-country institutions, thus missing opportunities to fund LMIC-led research.^23^ This echoes inequities in the wider newborn health and stillbirth prevention funding landscape.^7^, ^8^, ^9^, ^24^

Research funding represents approximately 4% of total aid funding mentioning newborn health.^24^ Yet, effective translation of scientific discovery to application in specific clinical contexts also requires evidence.^25^ Innovations are required across the pipeline to increase effective delivery of programmes funded by aid: elucidating causation, developing preventive and other interventions, and optimising context-appropriate implementation. In our analysis, preclinical and epidemiological studies were the most common, with only 10–20% of funding dedicated to testing interventions and less than 5% to implementation research, despite its widely recognised potential impact, especially in resource-constrained systems.^4^, ^12^

Stillbirth research funding was low in HICs and persistently so in LMICs over the 10-year period (2·5%); it represented 1·5% of global commitments by major funders in 2019–20, mirroring previous analyses of aid.^7^, ^8^, ^9^ If research and implementation investments continue in this trajectory, they are unlikely to support LMICs sufficiently to meet WHO's Every Newborn Action Plan target of fewer than 12 stillbirths per 1000 births.^4^, ^9^

Neonatal infections research received most (41·5%) LMIC-hosted funding between 2011 and 2020. Major funders’ recent research commitments also favoured preterm births and neonatal infections the most, yet the significant direct and indirect contributions of preterm births and neonatal infections to 2·3 million annual neonatal deaths suggests that research on scalable interventions remains insufficient.^26^, ^27^ In fact, grants related to preterm birth mostly supported basic science and preclinical studies, with most research addressing infections being observational. The focus on infections within newborn research is commensurate with the fact that neonatal infections cause 40% of neonatal deaths, yet previous analyses suggest newborns are grossly under-represented in infectious disease research funding.^26^ Although overall funding for research related to small, vulnerable newborns has increased over time (from 11·6% in 2011 to 15·4% in 2020), it remains less than a fifth of total funding available to LMIC recipients. This might be a key bottleneck for evaluating methods for preventing neonatal deaths (>30%) caused by prematurity, and for innovative delivery of safe care for preterm babies in resource-constrained health systems.^28^

This global, descriptive analysis of trends in, and categories of, research funding for newborn health and stillbirth combines the strengths of the most comprehensive and representative research funding database available (searching >6 million grants from >650 global funders), with in-depth content analysis by clinical researchers, maximising grant relevance and accurate categorisation. Tracking research and health financing is complex, and the Dimensions database has strengths and weaknesses in this regard. Global health research often involves multiple partners, and we apportioned funding accordingly, considering allocated funding by host country World Bank classification and region. However, indirect benefits and locations of unlisted recipients cannot be counted. Additionally, Dimensions and most funders do not provide data on grant distribution per recipient; however, our assumption of equal distribution is likely to have resulted in overestimation (rather than underestimation) of funding received by LMICs.

Dimensions translates, standardises, and updates data obtained from funders on a monthly basis, reducing missingness, double counting, and language or geographical bias; however, it inherently relies on transparent, accurate disclosures from funders,^18^ which might be less likely to be obtained, especially from for-profit biotechnology or pharmaceutical companies.^29^ Programmatic donors do not always delineate research-specific components in public data; for example, USAID is an important funder of embedded research whose financial contributions might be missing in a research database. However, we note that all major research donors for maternal and newborn health listed in a recent WHO-led publication are included.^13^ Grant descriptions that do not specify newborn health or stillbirth terms might not be counted or credited to funders. For a small proportion (5·1%) of included grants, monetary amounts were missing, probably resulting in under-representation of in-country contributions of certain UMIC national funders in particular (eg, in Brazil, South Africa, and Russia). Funding analyses typically consider total commitments; however, although it is useful to assess funder priorities (as per objective 1), this approach potentially overstates the funding available annually to researchers, since publicised amounts are usually dispersed over multiple years and partners. We accounted for this limitation in objective 2, by apportioning the 2011–20 total from all active grants (including commitments before 2011).

Targeted, sustainable, and equitably conducted research across the pipeline is one pillar of the strategy to advance newborn health and reduce stillbirths globally.^4^ Here, we mapped the research funding landscape to directly measure donor commitments to research and indirectly describe research activity itself. We did not compare funders’ disbursements versus commitments or the quantity or quality of recipients’ research outputs. Although we place our descriptive findings in the context of neonatal mortality and the stillbirth burden, further modelling of the impact of funding decisions on newborn outcomes or stillbirths will be needed to guide financing recommendations. Importantly, research funding is only effective alongside the strengthened research infrastructure required for high-quality outputs, including human resource planning and training, laboratory networks, unincumbered supply chains, and accessible ethical and regulatory approval processes; such activities are beyond the scope of most research grants (36 [1·8%] of 1985 LMIC awards in our data). Finally, the need for funder transparency is clear. The Dimensions platform could play a key role in improving publicly available data, by publishing reporting guidance for funders and tracking their provision of open data.

Tracking research investment is necessary for accountability, assessing whether health priorities with a large burden are receiving commensurate funding. Substantial funding is allocated for newborn health and stillbirth research, which we describe in detail by research topic, location, and study type. However, our findings highlight major disparities in funding allocation between HICs and LMICs, and very low proportions of funding allocated to stillbirth research in all contexts. These data make the case that reallocation of research funding could accelerate progress in regions where stillbirths and neonatal mortality risks and burdens are highest, notably for the most vulnerable born too small or too soon. Importantly, even for the inadequate amount of funding allocated to LMICs, most is still given via HICs. Directly investing in LMIC-led research could enable faster progress in locally led research to achieve off-track targets for neonatal survival and preventing stillbirths.

## Data sharing

Pooled summary tables generated will be deposited online at the time of publication at https://doi.org/10·17037/DATA.00003095 with data access subject to approval by collaborating parties. Analysed datasets and code are stored in the London School of Hygiene & Tropical Medicine repository and available upon reasonable request via email to the corresponding author.

## Declaration of interests

We declare no competing interests.

## Supplementary Materials

French translation of the abstract

German translation of the abstract

Spanish translation of the abstract
