# Supplementary material for: Research funding for newborn health and stillbirths, 2011–20: a systematic analysis of levels and trends
Source: Lancet Glob Health. 2023 Oct 17;11(11):e1794–804. doi: 10.1016/S2214-109X(23)00379-0 (PMC10603613; doi:10.1016/S2214-109X(23)00379-0)
Supplement: French translation of the abstract [file mmc1.pdf]

THE LANCET
Global Health

Supplementary appendix 1

This translation in French was submitted by the authors and we reproduce it as supplied. It has not been peer reviewed. *The Lancet*'s editorial processes have only been applied to the original in English, which should serve as reference for this manuscript.

Cette traduction en français a été proposée par les auteurs et nous l'avons reproduite telle quelle. Elle n'a pas été examinée par des pairs. Les processus éditoriaux du *Lancet* n'ont été appliqués qu'à l'original en anglais et c'est cette version qui doit servir de référence pour ce manuscrit.

Supplement to: Agravat P, Loucaides EM, Kumar MB, et al. Research funding for newborn health and stillbirths, 2011–20: a systematic analysis of levels and trends. *Lancet Glob Health* 2023; **11:** e1794–804.

Résumé

Introduction

Il est estimé que 4·4 millions de décès de nouveau-nés et de mortinaissances se sont produits dans le monde en 2020, et que 98 % de ces décès sont survenus dans des pays à faible revenu et à revenu intermédiaire (PRFI). Notre objectif était d'analyser les subventions pour la recherche sur les nouveau-nés et les mortinaissances accordées par les principaux bailleurs de fonds en 2019-20, ainsi que tous les fonds de recherche alloués aux institutions basées dans les PRFI en 2011-20.

Méthodologie

Pour cette analyse systématique, nous avons recherché dans Dimensions, la plus grande base de données de financement de la recherche au monde, des subventions pertinentes pour la recherche sur les nouveau-nés et les mortinaissances. Les subventions incluses ont été catégorisées par une analyse approfondie du contenu, avec des analyses quantitatives descriptives par pays bailleur de fonds et pays bénéficiaire, type d'étude, sujet et année.

Résultats

À l'échelle mondiale, en 2019-20, les principaux bailleurs de fonds ont accordé un total annuel moyen de 577·1 millions de dollars américains (US$) pour la recherche sur les nouveau-nés et les mortinaissances (total moyen de 550 subventions par an). Sur 577·1 millions de dollars, 166·3 millions de dollars (28·8%) ont été consacrés à la recherche sur les nouveau-nés de petite taille et vulnérables, mais seulement 8·4 millions de dollars (1·5%) ont été consacrés à la recherche sur les mortinaissances. La majorité des fonds, 537 millions de dollars (93%), ont été alloués à des organisations basées dans des pays à revenu élevé. Entre 2011 et 2020, les bénéficiaires basés dans les PRFI ont été nommés sur 1985 subventions de tous les bailleurs de fonds d'une valeur totale de 486·7 millions de dollars, dont 73·1 millions de dollars (15·0%) ont été alloués à la recherche sur les nouveau-nés petits et vulnérables et 12·0 millions de dollars (2-5%) ont été alloués à la recherche sur les mortinaissances. La plupart des fonds destinés aux PRFI ont soutenu des études pré-cliniques ou d'observation (236·8 millions de dollars [48·7%] sur 486·7 millions de dollars). La recherche de mise en œuvre des projets de santé n'ayant reçu que 13·9 millions de dollars (2·9%).

Interprétation

Bien que les investissements dans la recherche liée à la santé néonatale et à la mortinatalité aient augmenté entre 2011 et 2020, il existe des disparités marquées dans la distribution géographique, entre les principales causes de mortalité et entre les types de projets de recherche. La recherche sur les mortinaissances a reçu un financement minimal tant dans les pays à revenu élevé que dans les PRFI, malgré un nombre de décès similaire aux nouveau-nés. L'investissement direct dans la recherche menée par les PRFI, en particulier pour la recherche de mise en œuvre, pourrait accélérer la lenteur des progrès mondiaux en matière de prévention des mortinaissances et de survie des nouveau-nés.
