# Supplementary material for: Research funding for newborn health and stillbirths, 2011–20: a systematic analysis of levels and trends
Source: Lancet Glob Health. 2023 Oct 17;11(11):e1794–804. doi: 10.1016/S2214-109X(23)00379-0 (PMC10603613; doi:10.1016/S2214-109X(23)00379-0)
Supplement: German translation of the abstract [file mmc2.pdf]

THE LANCET
Global Health

Supplementary appendix 2

This translation in German was submitted by the authors and we reproduce it as supplied. It has not been peer reviewed. *The Lancet*'s editorial processes have only been applied to the original in English, which should serve as reference for this manuscript.

Diese Übersetzung in deutscher Sprache wurde von den Autoren eingereicht und wir reproduzieren sie wie vorgelegt. Die Übersetzung wurde nicht von Experten begutachtet. Die redaktionellen Prozesse von The Lancet wurden nur auf das Original in englischer Sprache angewendet, das als Referenz für dieses Manuskript dienen soll.

Supplement to: Agravat P, Loucaides EM, Kumar MB, et al. Research funding for newborn health and stillbirths, 2011–20: a systematic analysis of levels and trends. *Lancet Glob Health* 2023; **11:** e1794–804.

Zusammenfassung

Hintergrund

Im Jahr 2020 gab es weltweit schätzungsweise 4· 4 Millionen Tode im Neugeborenen-Alter und Totgeburten. 98 % dieser Todesfälle ereignen sich in Ländern mit niedrigem und mittlerem Einkommen (LMICs). Unser Ziel war es, neue Forschungszuschüsse für Neugeborene und Totgeburten zu analysieren, die von großen Geldgebern im Zeitraum 2019-20 vergeben wurden, sowie alle Forschungsmittel, die LMIC-basierten Einrichtungen im Zeitraum 2011-20 zugewiesen wurden.

Methoden

Für diese systematische Analyse durchsuchten wir Dimensions, die weltweit größte Datenbank für Forschungsförderung, nach Zuschüssen, die für die Forschung zu Neugeborenen und Totgeburten relevant sind. Eingeschlossene Förderungen wurden durch eine eingehende Inhaltsanalyse kategorisiert, mit deskriptiven quantitativen Analysen nach Geldgebern und Empfängerländern, Forschungspipeline, Thema und Jahr.

Ergebnisse

Weltweit haben die wichtigsten Geldgeber 2019-20 im Durchschnitt 577· 1 Millionen US-Dollar pro Jahr für die Forschung von Neugeborenen und Totgeburten bewilligt (insgesamt 550 Zuschüsse pro Jahr). Von den 577· 1 Mio. USD waren 166· 3 Mio. USD (28· 8%) für die Forschung an kleinen und gefährdeten Neugeborenen bestimmt, aber nur 8· 4 Mio. USD (1· 5%) für die Forschung an Totgeburten. Der Großteil der Forschungsförderung, 537· 0 Mio. USD (93· 0%), wurde Organisationen in Ländern mit hohem Einkommen zugewiesen. Zwischen 2011 und 2020 wurden durch 1985 Zuschüsse aller Geldgeber 486· 7 Mio. USD an Empfänger aus LMIC vergeben, von denen 73· 1 Mio. USD (15· 0%) für die Forschung an kleinen und gefährdeten Neugeborenen und 12· 0 Mio. USD (2· 5%) für die Forschung an Totgeburten bereitgestellt wurden. Die meisten LMIC-Fördermittel wurden für präklinische oder Beobachtungsstudien verwendet (236· 8 Mio. USD [48· 7%] von 486· 7 Mio. USD), während die Implementierungsforschung nur 13· 9 Mio. USD (2· 9 %) erhielt.

Interpretation

Obwohl die Investition in die Forschung für die Gesundheit von Neugeborenen und Verminderung der Totgeburten zwischen 2011 und 2020 zugenommen haben, gibt es deutliche Unterschiede in der geografischen Verteilung, zwischen Todesursachen und zwischen den verschiedenen Arten von Forschungsvorhaben. Die Forschung im Bereich der Totgeburten wurde sowohl in Ländern mit hohem Einkommen als auch in LMIC nur minimal finanziert, obwohl die Zahl der Todesfälle im Vergleich zu Neugeborenen ähnlich hoch ist. Direkte Investitionen in die von LMIC geleitete Forschung, insbesondere in die Umsetzungsforschung, könnten den langsamen globalen Fortschritt bei der Prävention von Totgeburten und dem Überleben von Neugeborenen beschleunigen.
