# Supplementary material for: Research funding for newborn health and stillbirths, 2011–20: a systematic analysis of levels and trends
Source: Lancet Glob Health. 2023 Oct 17;11(11):e1794–804. doi: 10.1016/S2214-109X(23)00379-0 (PMC10603613; doi:10.1016/S2214-109X(23)00379-0)
Supplement: Spanish translation of the abstract [file mmc3.pdf]

THE LANCET
Global Health

Supplementary appendix 3

This translation in Spanish was submitted by the authors and we reproduce it as supplied. It has not been peer reviewed. *The Lancet*'s editorial processes have only been applied to the original in English, which should serve as reference for this manuscript.

Los autores nos proporcionaron esta traducción al español y la reproducimos tal como nos fue entregada. No la hemos revisado. Los procesos editoriales de *The Lancet* se han aplicado únicamente al original en inglés, que debe servir de referencia para este manuscrito.

Supplement to: Agravat P, Loucaides EM, Kumar MB, et al. Research funding for newborn health and stillbirths, 2011–20: a systematic analysis of levels and trends. *Lancet Glob Health* 2023; **11:** e1794–804.

Resumen

Introducción

Se estima que en 2020 se produjeron 4·4 millones de muertes de recién nacidos y mortinatos en todo el mundo. El 98% de estas muertes se produjeron en países de ingresos bajos y medios (PIBM). Nuestro objetivo fue analizar las nuevas ayudas a la investigación de recién nacidos y mortinatos otorgadas por los principales financiadores en 2019-20, y todos los fondos para la investigación asignados a instituciones con sede en PIBM en 2011-20.

Métodos

Realizamos una búsqueda sistemática en Dimensions, la base de datos de financiación de proyectos de investigación más grande del mundo, de ayudas financieras relacionadas con la investigación neonatal y de mortinatos. Las ayudas incluídas se categorizaron empleando un análisis de contenido en profundidad, con análisis cuantitativos descriptivos por país financiador y país receptor, línea de investigación, tema y año.

Resultados

A nivel mundial, en 2019-20, los principales financiadores otorgaron una media total anual de 577·1 millones de dólares estadounidenses por año para la investigación de recién nacidos y mortinatos (media total de 550 ayudas al año). 166·3 millones de dólares (28·8%) de 577·1 millones de dólares se destinaron a la investigación de recién nacidos pequeños y vulnerables, pero solo 8·4 millones de dólares (1·5%) se destinaron a la investigación de mortinatos. La mayor parte de la financiación, 537·0 millones de dólares (93·0%), se concedió a organizaciones con sede en países de ingresos altos. Entre 2011 y 2020, los beneficiarios de los PIBM fueron nombrados en 1985 subvenciones de todos los financiadores por valor de 486·7 millones de dólares, de los cuales 73·1 millones (15·0%) se asignaron a la investigación de recién nacidos pequeños y vulnerables y 12·0 millones (2·5%) se destinaron a la investigación de mortinatos. La mayor parte de la financiación de los PIBM se destinó a estudios preclínicos o de observación (236·8 millones de dólares [48·7%] de 486·7 millones de dólares), mientras que las investigaciones sobre la implementación sólo recibieron 13·9 millones de dólares (2·9%).

Interpretación

Aunque la inversión en investigación relacionada con la salud neonatal y los mortinatos ha aumentado entre 2011 y 2020, existen marcadas disparidades en la distribución geográfica, entre las principales causas de mortalidad y entre los tipos de proyectos de investigación. La investigación sobre la mortinatalidad recibió una financiación mínima tanto en los países de ingresos altos como en los PIBM, a pesar de que el número de muertes es similar al de neonatos. La inversión directa en investigación dirigida por los PIBM, especialmente para la investigación de la implementación, podría acelerar el lento progreso mundial en la prevención de la mortinatalidad y la supervivencia neonatal.
