# Supplementary material for: Research funding for newborn health and stillbirths, 2011–20: a systematic analysis of levels and trends
Source: Lancet Glob Health. 2023 Oct 17;11(11):e1794–804. doi: 10.1016/S2214-109X(23)00379-0 (PMC10603613; doi:10.1016/S2214-109X(23)00379-0)
Supplement: Supplementary appendix 4 [file mmc4.pdf]

THE LANCET
Global Health

Supplementary appendix 4

This appendix formed part of the original submission and has been peer reviewed. We post it as supplied by the authors.

Supplement to: Agravat P, Loucaides EM, Kumar MB, et al. Research funding for newborn health and stillbirths, 2011–20: a systematic analysis of levels and trends. *Lancet Glob Health* 2023; **11:** e1794–804.

SUPPLEMENTARY APPENDIX

Research funding for newborn health and stillbirths: systematic analyses of levels and trends (2011-2020)

Priyesh Agravat[1*]MBBS, Eva M Loucaides[2*]PhD, Meghan Bruce Kumar[2,3]PhD, Anna Howells[4] MRCPCH, Alexandra Molina García[5]MPH, Ismail Sebina[6] PhD, Núria Balanza[7] MSc, Elizabeth J A Fitchett[2+]MPH, Joy E Lawn[2+] PhD

*Joint first author; +Joint senior authors

Affiliations:

1. St George's, University of London, London, UK
2. London School of Hygiene & Tropical Medicine, London, UK
3. KEMRI-Wellcome Trust, Nairobi, Kenya
4. King's College Hospital NHS Foundation Trust, London, UK
5. Centro de Salud San Fernando, Servicio Madrileño de Salud, Madrid, Spain
6. QIMR Berghofer Medical Research Institute, Herston, Queensland, Australia
7. ISGlobal, Hospital Clínic - Universitat de Barcelona, Barcelona, Spain

Table of Contents

1. The Dimensions database 2
2. Literature search 18
3. Search strategy used to search the Dimensions database 19
4. Table S1. Categorisation of included grants by thematic area of the described research activity 20
5. Table S2. Categorisation of included grants by research pipeline 22
6. Illustrative examples of included grants and categorisation 23
7. Table S3. List of funders of included grants 27
8. Table S4. Summary of total new funding awarded per year by major funders (mean, 2019-2020) 30
9. Table S5. All funding received for research related to newborn health or stillbirths via grants with at least one recipient organization in a low- or middle-income country, active between 2011 and 2020 33
10. Figure S1. Heatmaps of funding (USD) awarded in 2019 and 2020 from major funders, by research theme and research pipeline categories across SDG super region 35
11. Table S6. Total funding (USD) active between 2011-2020 awarded to at least one LMIC recipient, by Year and by Thematic Area, Pipeline, Funders and Recipients 36
12. Table S7. Summary of mean total new funding awarded per year by all funders in grants with at least one LMIC-hosted recipient organization (mean 2019-2020) 37
13. Table S8. Total funding (USD) active between 2011-2020 awarded to at least one LMIC recipient, Number of Active Grants and Median Available Funding per Grant (USD) and Interquartile range (IQR), by Recipients and Funder 40
14. Figure S2. Bubble graph demonstrating relationship between neonatal mortality burden (mean annual neonatal deaths per 1000 live births) and total research investments received by LMIC institutions from 2011-2020. 43
15. Figure S3. Newborn health and stillbirth research funding trends over time, and by thematic area 44
16. References 45

1. The Dimensions database

- Dimensions is an interlinked research information system provided by Digital Science https://www.dimensions.ai/
- It uses automated AI-supported methods to collate funding data from open-access web-based sources and is updated on a monthly basis
- It is not actively restricted to English, and lists automatic translation of abstracts when provided in another language
- The database allows the use of keywords and Boolean search terms to identify relevant grants, similar to online databases of scientific publications
- At the time of publication, the database includes more than 7 million grants worth more than 2.3 trillion USD from 669 funders worldwide
- Available grant information includes the title, abstract, funding amount, start and end dates, and the name and location of funders and recipients
- As Dimensions sources data from funder platforms it lists only a single funder for each grant but can list multiple recipients
- Grant funding amounts are obtained in their original currencies as well as converted to US dollars, on the basis of the exchange rate at the time of the start date of the grant; in the case that a yearly distribution of the funding amount is provided (eg US National Institutes of Health projects), the funding amount is converted for each year's exchange rate
- The 672 funders of grants listed in the Dimensions database as of June 2023 are provided below[1]:

Funding Institution	Host country
Academy of Finland (AKA)	Finland
Academy of Medical Sciences(AMS)	United Kingdom
Action Medical Research (Action Medical Research)	United Kingdom
Administration for Children and Families(ACF)	United States
Administration for Community Living (ACL)	United States
Advanced Research Projects Agency-Energy (ARPA-E)	United States
Agence Nationale de la Recherche(ANR)	France
Agencia Nacional de Investigación y Desarrollo (ANID)	Chile
Agency for Healthcare Research and Quality (AHRQ)	United States
Agency for Toxic Substances And Disease Registry (ATSDR)	United States
Agricultural Research Service (ARS)	United States
Agroscope (ALP (FAM))	Switzerland
Alberta Cancer Foundation (AlbertaCancer Foundation)	Canada
Alberta Centre for Child, Family and Community Research (ACCFCR)	Canada
Alberta Innovates (AB Innov)	Canada
Alcohol Research UK (Alcohol Research UK)	United Kingdom
Alex's Lemonade Stand Foundation (ALSF)	United States
Alfred P. Sloan Foundation	United States
Alopecia UK (AUK)	United Kingdom
Alzheimer Forschung Initiative (AFI)	Germany
Alzheimer Nederland (ISAO)	Netherlands
Alzheimer Society ofCanada(ASC)	Canada

[1] a small number of funders may have been added after the last search included in this review

**Funding Institution**	**Host country**
Alzheimer's Association (ALZ)	United States
Alzheimer's Drug Discovery Foundation (ADDF)	United States
Alzheimer's Society (Alzheimer'sSociety)	United Kingdom
Alzheimer’s Research UK (ARUK)	United Kingdom
American Association For Cancer Research (AACR)	United States
American Diabetes Association (ADA)	United States
American Epilepsy Society (AES)	United States
American Federation for Aging Research (AFAR)	United States
American Foundation for Suicide Prevention (AFSP)	United States
American Heart Association (AHA)	United States
American Institute for Cancer Research (AICR)	United States
American Parkinson Disease Association (APDA)	United States
American Society for Radiation Oncology (ASTRO)	United States
Andrew McDonough B+Foundation (The Andrew McDonough B+Foundation)	United States
Animal Cancer Foundation (ACF)	United States
Anthony Nolan (Anthony Nolan)	United Kingdom
Antibiotic Research UK (ANTRUK)	United Kingdom
Anticancer Fund (Anticancer Fund)	Belgium
Arcadia Fund (Arcadia)	United Kingdom
Arnold and Mabel Beckman Foundation (Beckman)	United States
Arthritis Foundation (AF)	United States
Arthritis Society (Arthritis Society)	Canada
Arts And Humanities Research Council (AHRC)	United Kingdom
Asthma And Lung UK (Asthma UK)	United Kingdom
Ataxia UK (AtaxiaUK)	United Kingdom
Attorney-General's Department Australia(AGDA)	Australia
Auckland Medical Research Foundation (AMRF)	New Zealand
Australian Centre For International Agricultural Research (ACIAR)	Australia
Australian Communications And Media Authority (ACMA)	Australia
Australian Federal Police(AFP)	Australia
Australian Renewable Energy Agency (ARENA)	Australia
Australian Research Council (ARC)	Australia
Australian Taxation Office(ATO)	Australia
Australian Trade And Investment Commission (Austrade)	Australia
Austrian Research Promotion Agency (FFG)	Austria
Autism Science Foundation (ASF)	United States
Autism Speaks (Autism Speaks)	United States
Autistica (Autistica)	United Kingdom
Bank of Sweden Tercentenary Foundation (RJSE)	Sweden
Batten Disease Support and Research Association (BDSRA)	United States
Be Strong, Fight On! (BSFO)	United States
Bear Necessities Pediatric Cancer Foundation (BN)	United States
Belgian Federal Science Policy Office (BELSPO)	Belgium
Bill & Melinda Gates Foundation (BMGF)	United States
Biotechnology and Biological Sciences Research Council (BBSRC)	United Kingdom
Bladder Cancer Advocacy Network (BCAN)	United States

Funding Institution	Host country
Blood Cancer UK (Bloodwise)	United Kingdom
Bone Cancer Research Trust (BCRT)	United Kingdom
Bowel Cancer UK (CCC)	United Kingdom
Bowel Research UK (BDRF)	United Kingdom
BRACE (BRACE)	United Kingdom
Bradens' Hope For Childhood Cancer (BHCC)	United States
Brain & Behavior Research Foundation	United States
Brain Aneurysm Foundation (BAF)	United States
Brain Canada Foundation	Canada
Brain Research UK (BRT)	United Kingdom
Brain Tumour Charity (Brain Tumour Charity)	United Kingdom
Brain Tumour Foundation of Canada(Brain Tumour Foundation of Canada)	Canada
Brain Tumour Research (Brain Tumour Research)	United Kingdom
Breast Cancer Now (BCN)	United Kingdom
Breast Cancer Society of Canada (BCSC)	Canada
British Academy (BA)	United Kingdom
British Association for Counselling And Psychotherapy (BACP)	United Kingdom
British Council For Prevention of Blindness (BCPB)	United Kingdom
British Heart Foundation (BHF)	United Kingdom
British Journal of Anaesthesia (BJA)	United Kingdom
British Scoliosis Research Foundation (BSRF)	United Kingdom
British Sjögren's Syndrome Association (BSSA)	United Kingdom
British Skin Foundation (BSF)	United Kingdom
Brittle Bone Society (BBS)	United Kingdom
Burroughs Wellcome Fund (BWF)	United States
C17 Council (C17)	Canada
California Breast Cancer Research Program (CBCRP)	United States
California HIV/AIDS Research Program (CHRP)	United States
California Institute for Regenerative Medicine (CIRM)	United States
Canada Foundation for Innovation (CFI)	Canada
Canada-California Strategic Innovation Partnership (CCSIP)	United States
Canadian Association of Radiation Oncology (CARO)	Canada
Canadian Breast Cancer Foundation (Canadian Breast Cancer Foundation)	Canada
Canadian Cancer Society (CCS)	Canada
Canadian Institutes of Health Research (CIHR)	Canada
Canadian Partnership Against Cancer (Canadian Partnership Against Cancer)	Canada
Canadian Prostate Cancer Research Initiative (CPCRI)	Canada
Canadian Sleep & Circadian Network (CSCN)	Canada
Canadian Tobacco Control Research Initiative (CTCRI)	Canada
Cancer Australia(CA)	Australia
Cancer Care Nova Scotia (CCNS)	Canada
Cancer Care Ontario (CCO)	Canada
Cancer Institute of New South Wales (CINSW)	Australia
Cancer Prevention and Research Institute Texas (CPRIT)	United States
Cancer Research Society (SRC)	Canada
Cancer Research UK (CRUK)	United Kingdom

Funding Institution	Host country
Cancer Research Wales (CRW)	United Kingdom
Cancer Care Manitoba (CCMB)	Canada
Cancer Free KIDS	United States
Carson Leslie Foundation (CLF)	United States
Center for Information Technology (CIT)	United States
Center for Neuroscience and Regenerative Medicine (CNRM)	United States
Center for Scientific Review (CSR)	United States
Centers for Disease Control and Prevention (CDC)	United States
Centers for Medicare and Medicaid Services (CMS)	United States
Central Norway Regional Health Authority	Norway
Chest Heart and Stroke Scotland (CHSS)	United Kingdom
Chief Scientist Office (CSO)	United Kingdom
Childhood Eye Cancer Trust (CHECT)	United Kingdom
Children with Cancer UK (ChildrenwithCancerUK)	United Kingdom
Children's Cancer and Leukaemia Group (CCLG)	United Kingdom
Children's Tumor Foundation (CTF)	United States
Children's Liver Disease Foundation (CLDF)	United Kingdom
Chronic Disease Research Foundation (CDRF)	United Kingdom
Coeliac UK (TCS)	United Kingdom
Combat Casualty Care Research Program (CCCRP)	United States
Commonwealth Fund (TCF)	United States
Congressionally Directed Medical Research Programs (CDMRP)	United States
Coordenação de Aperfeiçoamento de Pessoal de Nível Superior (CAPES)	Brazil
Council for Grants of the President of the Russian Federation (PGF)	Russia
Council for International Exchange of Scholars (CIES)	United States
Craig H Neilsen Foundation (CHN)	United States
Croatian Science Foundation (HRZZ)	Croatia
Crohn's and Colitis Foundation (CCF)	United States
Crohn's and Colitis UK (Crohn's and ColitisUK)	United Kingdom
Cure Alzheimer’s Fund (CAF)	United States
CURE Childhood Cancer (CUREChildhoodCancer)	United States
CURE Epilepsy (CURE)	United States
Cure Parkinson's Trust (CureParkinson'sTrust)	United Kingdom
Cure PSP	United States
Cure Search for Children's Cancer	United States
Cystic Fibrosis Trust (CF)	United Kingdom
Czech Academy of Sciences (AVČR)	Czechia
Czech Office for Surveying, Mapping and Cadastre (ČÚZK)	Czechia
Czech Science Foundation (GAČR)	Czechia
Dalle Molle Institute for Artificial Intelligence Research (IDSIA)	Switzerland
Dam Foundation	Norway
Damon Runyon Cancer Research Foundation (DRCRF)	United States
Danish Agency for Science and Higher Education (DASHE)	Denmark
Danish Ministry of Higher Education and Science (UFM)	Denmark
Debra (Debra)	United Kingdom
Defense Advanced Research Projects Agency (DARPA)	United States

Funding Institution	Host country
Defense Logistics Agency (DLA)	United States
DefenseThreat Reduction Agency (DTRA)	United States
Department for Environment, Food and Rural Affairs (DEFRA)	United Kingdom
Department of Agriculture, Water And the Environment (AWE)	Australia
Department of Biotechnology (DBT)	India
Department of Defence (DoD-Au)	Australia
Department of Education, Skills and Employment (DET-Au)	Australia
Department of Finance (DoF-Au)	Australia
Department of Foreign Affairs and Trade (DFAT)	Australia
Department of Health (DoH-Au)	Australia
Department of Home Affairs (DHA-Au)	Australia
Department of Industry, Science, Energy and Resources (DIIS)	Australia
Department of Infrastructure, Transport, Regional Development and Communications (DIRDC)	Australia
Department of Science and Technology (DST)	India
Department of Social Services (DSS)	Australia
Department of the Prime Minister and Cabinet (PM&C)	Australia
Department of the Treasury (DoT-Au)	Australia
Department of Veterans Affairs (DVA)	Australia
Deutsche Forschungsgemeinschaft (DFG)	Germany
Diabetes Research and Wellness Foundation (DRWF)	United Kingdom
Diabetes UK (Diabetes UK)	United Kingdom
Directorate for Biological Sciences (NSFBIO)	United States
Directorate for Computer & Information Science & Engineering (NSFCISE)	United States
Directorate for Engineering (NSFENG)	United States
Directorate for Geosciences (NSFGEO)	United States
Directorate for Mathematical & Physical Sciences (NSFMPS)	United States
Directorate for Social, Behavioral & Economic Sciences (NSFSBE)	United States
Directorate for STEM Education (EDU)	United States
Directorate for Technology, Innovation and Partnerships (NSFTIP)	United States
Domestic Nuclear Detection Office (DNDO)	United States
Duchenne UK (DUK)	United Kingdom
Dunhill Medical Trust (DMT)	United Kingdom
Dutch Cancer Society (DCS)	Netherlands
Dutch Research Council (NWO)	Netherlands
Economic and Social Research Council (ESRC)	United Kingdom
Economic Research Service (ERS)	United States
EEA and Norway Grants (EEA Grants)	Belgium
Einstein Healthcare Network (AEHN)	United States
Elaine Roberts Foundation (ERF)	United States
Elrha (Elrha)	United Kingdom
Engineering and Physical Sciences Research Council (EPSRC)	United Kingdom
Environmental Protection Agency (EPA)	United States
Epilepsy Action (Epilepsy Action)	United Kingdom
Epilepsy Research UK (ERUK)	United Kingdom
Estonian Research Council (ETAg)	Estonia
Eunice Kennedy Shriver National Institute of Child Health and Human Development (NICHD)	United States

**Funding Institution**	**Host country**
European & Developing Countries Clinical Trials Partnership (EDCTP)	Netherlands
European Commission (EC)	Belgium
European Cooperation in Science and Technology (COST)	Belgium
European Molecular Biology Organization (EMBO)	Germany
European Organisation for Research and Treatment of Cancer (EORTC)	Belgium
European Research Council (ERC)	Belgium
European School of Materials (EUSMAT)	Germany
Federal Department of Defence, Civil Protection and Sports (DDPS)	Switzerland
Federal Department of Economic Affairs Education and Research (EAER)	Switzerland
Federal Department of Environment, Transport, Energy and Communications (DETEC)	Switzerland
Federal Department of Finance (SIF)	Switzerland
Federal Department of Foreign Affairs (FDFA)	Switzerland
Federal Department of Home Affairs (FDHA)	Switzerland
Federal Department of Justice and Police (FDJP)	Switzerland
Federal Food Safety and Veterinary Office (FSVO)	Switzerland
Federal Government of Nigeria (tetfund)	Nigeria
Federal Ministry for Digital and Transport (BMDV)	Germany
Federal Ministry for Economic Affairs and ClimateAction (BMWK)	Germany
Federal Ministry for the Environment, Nature Conservation, Nuclear Safety and Consumer Protection (BrMmUanVy)	Germany
Federal Ministry of Education and Research (BMBF)	Germany
Federal Ministry of Food and Agriculture (BMEL)	Germany
Federal Office for the Environment (BAFU)	Switzerland
Federal Office of Public Health (FOPH)	Switzerland
Federal Roads Office (ASTRA)	Switzerland
Federal Social Insurance Office (BSV)	Switzerland
Fight for Sight (Fight for Sight)	United Kingdom
Financial Markets Foundation for Children (FMFFC)	Australia
Fisheries Research and Development Corporation (FRDC)	Australia
Flashes of Hope (FlashesofHope)	United States
Fogarty International Center (FIC)	United States
Fondation ARC pour la Recherche sur le Cancer (Fondation ARC pour la Recherche sur le Cancer)	France
Fondation Botnar (FBO)	Switzerland
Fondation de France	France
Fondation Leducq (Leducq)	France
Fondation Vaincre Alzheimer (LECMA)	France
Fonds de Recherche du Québec -Santé (FRQS)	Canada
Fonds de Recherche du Québec -Société et Culture (FRQSC)	Canada
Fonds de Recherche du Québec – Nature et Technologies (FRQNT)	Canada
Fonds National de la Recherche (FNR)	Luxembourg
Ford Foundation (Ford)	United States
Foundation Fighting Blindness (FFB)	United States
Foundation for Baltic and East European Studies	Sweden
Foundation for Polish Science (FNP)	Poland
French National Cancer Institute (INCA)	France
Friends of Rosie Children's Cancer Research Fund (FORC)	United Kingdom

Funding Institution	Host country
Fritz Thyssen Foundation	Germany
Fund for Scientific Research (FRS FNRS)	Belgium
Fundação Carlos Chagas Filho de Amparo à Pesquisa do Estado do Rio de Janeiro (FAPERJ)	Brazil
Fundação para a Ciênciae Tecnologia (FCT)	Portugal
FWF Austrian Science Fund (FWF)	Austria
Garnett Passe and Rodney Williams Memorial Foundation (GPRWMF)	Australia
Genome Canada (Genome Canada)	Canada
Geoscience Australia (GA)	Australia
Gerber Foundation (Gerber)	United States
German Association of Joint Industrial Applied Research Institutes (AIF)	Germany
Global Lyme Alliance (GLA)	United States
Government of the Czech Republic (VLÁDA)	Czechia
Goyder Institute for Water Research (GIWR)	Australia
Graham Boeckh Foundation (GBF)	Canada
Grains Research and Development Corporation (GRDC)	Australia
Grand Challenges Canada (GCC)	Canada
Great Barrier Reef Marine Park Authority (GBRMPA)	Australia
Great Ormond Street Hospital Children's Charity (Great Ormond Street Hospital Children'sCharity)	United Kingdom
Gulf of Mexico Research Initiative (GoMRI)	United States
Guts UK (GutsUK)	United Kingdom
Guy's and St Thomas' Charity (Guy's and St Thomas' Charity)	United Kingdom
Hans Böckler Foundation (HBS)	Germany
Health and Care Research Wales (NISCHR)	United Kingdom
Health Foundation	United Kingdom
Health Research(HRI)	United States
Health Research Board (HRB)	Ireland
Health Research Council of New Zealand (HRC)	New Zealand
Health Resources and Services Administration (HRSA)	United States
Healthcare Infection Society (HIS)	United Kingdom
Heart and Stroke Foundation (HSF)	Canada
Heart Research UK (HeartResearchUK)	United Kingdom
Henry Smith Charity (hsc)	United Kingdom
Hermon Slade Foundation (HSF)	Australia
Hewlett Foundation	United States
Horticulture Innovation Australia (HIA)	Australia
Hradec Králové Region (KHK)	Czechia
Hungarian Academy of Sciences (MTA)	Hungary
Hungarian Scientific Research Fund (OTKA)	Hungary
I CareI Cure Childhood Cancer Foundation (ICIC)	United States
Indian Council of Medical Research (ICMR)	India
Indian Health Service (IHS)	United States
Innosuisse–SwissInnovation Agency (INNOSUISSE)	Switzerland
Innovate UK (InnovateUK)	United Kingdom
Innovation and Technology Commission (ITC)	China
Innovation Fund Denmark (IFD)	Denmark
Inserm (INSERM)	France

Funding Institution	Host country
Institute for Evaluation of Labour Market and Education Policy (IFAU)	Sweden
Institute of Alcohol Studies (IAS)	United Kingdom
Institute of Museum and Library Services (IMLS)	United States
Instituto de Salud CarlosIII (ISCIII)	Spain
International Atomic Energy Agency (IAEA)	Austria
International Diabetes Federation (IDF)	Belgium
International Foundation for Science (IFS)	Sweden
International Human Frontier Science Program Organization (HFSP)	France
International Visegrad Fund (IVF)	Slovakia
Irish Cancer Society (Irish CancerSociety)	Ireland
Irish Research Council (IRC)	Ireland
Israel Science Foundation (ISF)	Israel
Italian Association for Cancer Research (AIRC)	Italy
James S. McDonnell Foundation (JSMF)	United States
Japan Agency for Medical Research and Development (AMED)	Japan
Japan Science and Technology Agency (JST)	Japan
Japan Society for the Promotion of Science (JSPS)	Japan
Joey's Wings (Joey'sWingsFoundation)	United States
John Templeton Foundation (Templeton)	United States
Juvenile Diabetes Research Foundation (JDRFUK)	United Kingdom
Juvenile Diabetes Research Foundation (JDRF)	United States
Karlovy Vary Region (KKV)	Czechia
Khalifa University of Science and Technology (KUSTAR)	United Arab Emirates
Kidney Research UK (Kidney Research UK)	United Kingdom
Kidscan Children's Cancer Research (Kidscan Children's Cancer Research)	United Kingdom
Klingenstein Third Generation Foundation (KTGF)	United States
Knut and Alice Wallenberg Foundation (KAW)	Sweden
Leukaemia & Lymphoma NI (Leukaemia&LymphomaNI)	United Kingdom
Leukaemia & Myeloma Research UK (Leukaemia&MyelomaResearchUK)	United Kingdom
Leukaemia UK (LeukaemiaUK)	United Kingdom
Leukemia & Lymphoma Society of Canada (LLSC)	Canada
Leukemia and Lymphoma Society (LLS)	United States
Leverhulme Trust (leverhulme)	United Kingdom
Liberec Region (KLI)	Czechia
Lister Institute of Preventive Medicine (ListerInstituteofPreventiveMedicine)	United Kingdom
Lullaby Trust (FSID)	United Kingdom
Lung Cancer Research Foundation (LCRF)	United States
LUN Gevity Foundation (LUNG)	United States
Macmillan Cancer Support (Macmillan Cancer Support)	United Kingdom
Macular Society (MacularSociety)	United Kingdom
Marie Curie(MC)	United Kingdom
Medical Research Council (MRC)	United Kingdom
Medical Research Foundation (Medical Research Foundation)	United Kingdom
Medical Research Scotland (SHERT)	United Kingdom
Melanoma Research Alliance (MRA)	United States
Meningitis Now (MeningitisNow)	United Kingdom

Funding Institution	Host country
Meningitis Research Foundation (MeningitisResearch Foundation)	United Kingdom
Mental Health Commission (MHC)	Australia
Mental Health Research Canada(MHRC)	Canada
Mesothelioma Applied Research Foundation (MARF)	United States
MIB Agents (MIBA)	United States
Michael Smith Health Research BC (MSFHR)	Canada
Ministère des Solidarités et de la Santé (DGOS)	France
Ministry of Agriculture (eAGRI)	Czechia
Ministry of Agriculture and Rural Development (MRiRW)	Poland
Ministry of Agriculture, Food and Rural Affairs (OMAFRA)	Canada
Ministry of Business, Innovation and Employment (MBIE)	New Zealand
Ministry of Colleges and Universities (Ministry of Colleges and Universities)	Canada
Ministry of Culture (MKČR)	Czechia
Ministry of Defence (MOCR)	Czechia
Ministry of Earth Sciences (MoES)	India
Ministry of Education and Research (HM)	Estonia
Ministry of Education and Science	Latvia
Ministry of Education Youth and Sports (MSMT)	Czechia
Ministry of Education, Science, Research and Sport of the Slovak Republic (MŠVVaŠ SR)	Slovakia
Ministry of Education, Universities and Research (MIUR)	Italy
Ministry of Foreign Affairs (MFACR)	Czechia
Ministry of Health (MZ)	Czechia
Ministry of Health Labour and Welfare (MHLW)	Japan
Ministry of Industry and Trade (MPO)	Czechia
Ministry of Justice (MS)	Czechia
Ministry of Labour and Social Affairs (MoLSA)	Czechia
Ministry of Regional Development (MMR)	Czechia
Ministry of Science and Higher Education (MNiSW)	Poland
Ministry of the Environment	Japan
Ministry of the Environment of the Czech Republic (MŽP)	Czechia
Ministry of the Interior (MV)	Czechia
Ministry of Transport (MD)	Czechia
Missile Defense Agency (MDA)	United States
Mitacs	Canada
MND Scotland	United Kingdom
Moorfields Eye Charity (MEC)	United Kingdom
Motor Neurone Disease Association (MND)	United Kingdom
MQ: Transforming Mental Health (MQ)	United Kingdom
Multiple Sclerosis Society (MS)	United Kingdom
Multiple System Atrophy Trust (Multiple System Atrophy Trust)	United Kingdom
Muscular Dystrophy UK (Muscular Dystrophy UK)	United Kingdom
Myeloma UK (MyelomaUK)	United Kingdom
Myrovlytis Trust (MyrovlytisTrust)	United Kingdom
National Aeronautics and Space Administration (NASA)	United States
National Agriculture and Food Research Organization (NARO)	Japan
National Archives and Records Administration (NARA)	United States

Funding Institution	Host country
National Blood Authority (NBA)	Australia
National Breast Cancer Foundation (NBCF)	Australia
National Cancer Centre (NCC)	Japan
National Cancer Institute (NCI)	United States
National Center for Advancing Translational Sciences (NCATS)	United States
National Center for Chronic Disease Prevention and Health Promotion (NCCDPHP)	United States
National Center for Complementary and Integrative Health (NCCIH)	United States
National Center for Emerging and Zoonotic Infectious Diseases (NCEZID)	United States
National Center for Environmental Health (NCEH)	United States
National Center for Health Statistics (NCHS)	United States
National Center for HIV/AIDS Viral Hepatitis STD and TB Prevention (NCHSTP)	United States
National Center for Immunization and Respiratory Diseases (NIP)	United States
National Center for Injury Prevention and Control (NCIPC)	United States
National Center on Birth Defects and Developmental Disabilities (NCBDD)	United States
National Centre for Research and Development (NCRD)	Poland
National Centre for the Replacement Refinement and Reduction of Animals in Research (NC3Rs)	United Kingdom
National Council for Scientific and Technological Development (CNPq)	Brazil
National Endowment for the Humanities (NEH)	United States
National Energy Technology Laboratory (NETL)	United States
National Eye Institute (NEI)	United States
National Eye Research Centre (National Eye Research Centre)	United Kingdom
National Geospatial-Intelligence Agency (NIMA)	United States
National Health and Medical Research Council (NHMRC)	Australia
National Heart Lung and Blood Institute (NHLBI)	United States
National Human Genome Research Institute (NHGRI)	United States
National Information Processing Institute (OPI)	Poland
National Institute for Health and Care Research (NIHR)	United Kingdom
National Institute for Occupational Safety and Health (NIOSH)	United States
National Institute of Allergy and Infectious Diseases (NIAID)	United States
National Institute of Arthritis and Musculoskeletal and Skin Diseases (NIAMS)	United States
National Institute of Biomedical Imaging and Bioengineering (NIBIB)	United States
National Institute of Dental and Craniofacial Research (NIDCR)	United States
National Institute of Diabetes and Digestive and Kidney Diseases (NIDDK)	United States
National Institute of Environmental Health Sciences (NIEHS)	United States
National Institute of Food and Agriculture (NIFA)	United States
National Institute of General Medical Sciences (NIGMS)	United States
National Institute of Justice (NIJ)	United States
National Institute of Mental Health (NIMH)	United States
National Institute of Neurological Disorders and Stroke (NINDS)	United States
National Institute of Nursing Research (NINR)	United States
National Institute of Standards and Technology (NIST)	United States
National Institute on Aging (NIA)	United States
National Institute on Alcohol Abuse and Alcoholism (NIAAA)	United States
National Institute on Deafness and Other Communication Disorders (NIDCD)	United States
National Institute on Disability, Independent Living, and Rehabilitation Research (NIDILRR)	United States
National Institute on Drug Abuse (NIDA)	United States

Funding Institution	Host country
National Institute on Minority Health and Health Disparities (NIMHD)	United States
National Institutes of Health Clinical Center (CLC)	United States
National Natural Science Foundation of China (NSFC)	China
National Oceanic and Atmospheric Administration (NOAA)	United States
National Pancreas Foundation (NPF)	United States
National Psoriasis Foundation (NPF)	United States
National Research Council (CNR)	Italy
National Research Foundation (NRF)	South Africa
National Science Board (NSFNSB)	United States
National Science Center (NCN)	Poland
National Science Foundation (NSF)	United States
National Security Agency (NSA)	United States
National Security Authority (NBÚ)	Czechia
Natural Environment Research Council (NERC)	United Kingdom
Natural Sciences and Engineering Research Council (NSERC)	Canada
Netherlands Enterprise Agency (RVO)	Netherlands
Netherlands Organisation for Health Research and Development (ZonMw)	Netherlands
Neurosciences Research Foundation (NRF)	United Kingdom
New Brunswick Health Research Foundation (NBHRF)	Canada
New Energy and Industrial Technology Development Organization (NEDO)	Japan
New York State Energy Research and Development Authority (NYSERDA)	United States
New York Stem Cell Foundation (NYSCF)	United States
NIHR Academy (TCC)	United Kingdom
NIHR Central Commissioning Facility (CCF)	United Kingdom
NIHR Evaluation Trials and Studies Coordinating Centre (NETS)	United Kingdom
Noah's Light Foundation (NLF)	United States
Nord Forsk (NordForsk)	Norway
North Carolina Biotechnology Center (NCBiotech)	United States
North West Cancer Research (NWCR)	United Kingdom
Northern Ireland Chest Heart and Stroke (Northern Ireland Chest Heart and Stroke)	United Kingdom
Northern Norway Regional Health Authority	Norway
NovaScotia Health Research Foundation (NSHRF)	Canada
Novo Nordisk Foundation (NNF)	Denmark
Office of Advanced Scientific Computing Research (ASCR)	United States
Office of Basic Energy Sciences (BES)	United States
Office of Biological and Environmental Research (BER)	United States
Office of Budget, Finance and Award Management (NSF BFA)	United States
Office of Fusion Energy Sciences (FES)	United States
Office of Information and Resource Management (NSF OIRM)	United States
Office of Inspector General (OIG)	United States
Office of Nuclear Energy (NE)	United States
Office of Nuclear Physics (NP)	United States
Office of Public Health Preparedness and Response (OPHPR)	United States
Office of Science (DOESC)	United States
Office of the Director (NSF OD)	United States
Office of the Director (OD)	United States

Funding Institution	Host country
Office of the National Coordinator for Health Information Technology (ONC)	United States
Office of the Secretary of Defense (OSD)	United States
Oncology Nursing Society (ONS)	United States
One Mind (OMI)	United States
Ontario Institute for Cancer Research (OICR)	Canada
Oracle Cancer Trust (OCT)	United Kingdom
Organ and Tissue Authority (OTA)	Australia
Orthopaedic Research (Orthopaedic Research)	United Kingdom
Orthopaedic Research and Education Foundation (OREF)	United States
Out of Zion (Hope4ATRT)	United States
Ovarian Cancer Action (OCA)	United Kingdom
Ovarian Cancer Canada (NOCA)	Canada
Ovarian Cancer Research Alliance (OCRA)	United States
Pancreatic Cancer Action Network (PCAN)	United States
Pancreatic Cancer Canada Foundation (PCCF)	Canada
Pancreatic Cancer Research Fund (Pancreatic Cancer Research Fund)	United Kingdom
Pancreatic Cancer UK (PancreaticCancerUK)	United Kingdom
Parkinson's Foundation (PDF)	United States
Parkinson's UK (Parkinson'sUK)	United Kingdom
Patient-Centered Outcomes Research Institute (PCORI)	United States
Pediatric Brain Tumor Foundation (PediatricBrainTumorFoundation)	United States
Pediatric Dermatology Research Alliance (PeDRA)	United States
Pediatric Oncology Group (POG)	Canada
Pershing Square Foundation (PSF)	United States
Pew Charitable Trusts (PewCharitableTrusts)	United States
Pharmacy Research UK (PharmacyResearchUK)	United Kingdom
Policy Innovation and Co-ordination Office (PICO)	China
Polish Academy of Sciences (PAN)	Poland
PROCURE (PROCURE)	Canada
Prostate Cancer Research (PCRC)	United Kingdom
Prostate Cancer UK (ProstateCancerUK)	United Kingdom
Psoriasis Association (PsoriasisAssociation)	United Kingdom
Public Health Agency (PHA)	United Kingdom
Pulmonary Fibrosis Foundation (PFF)	United States
Qatar National Research Fund (QNRF)	Qatar
Quebec Breast Cancer Foundation (QBCF)	Canada
Ragnar Söderberg Foundation (Söderberg)	Sweden
Research Corporation for Science Advancement (RCSA)	United States
Research Foundation -Flanders(FWO)	Belgium
Research Manitoba(MHRC)	Canada
Research Nova Scotia (Research NovaScotia)	Canada
Rheumatology Research Foundation (RheumatologyResearch Foundation)	United States
Robert Wood Johnson Foundation (RWJF)	United States
Roy Castle Lung Cancer Foundation (RoyCastleLungCancerFoundation)	United Kingdom
Royal College of Anaesthetists (RCoA)	United Kingdom
Royal Hospital for Neuro-disability (Royal Hospital forNeuro-disability)	United Kingdom

Funding Institution	Host country
Royal National Institute for Deaf People (RNID)	United Kingdom
Royal Osteoporosis Society (ROS)	United Kingdom
Royal Society (Royal Society)	United Kingdom
Royal Society of New Zealand (RSNZ)	New Zealand
Russian Foundation for Basic Research (RFBR)	Russia
Russian Science Foundation (RSF)	Russia
Safe Work Australia (SFA)	Australia
Sammy's Superheroes (SAMMYS)	United States
Sands (SPDA)	United Kingdom
São Paulo Research Foundation (FAPESP)	Brazil
Sarcoma Foundation of America(SFA)	United States
Sarcoma UK (SarcomaUK)	United Kingdom
Saskatchewan Cancer Agency (Saskatchewan Cancer Agency)	Canada
Saskatchewan Health Research Foundation (SHRF)	Canada
Schweizerische Bundeskanzlei (BK)	Switzerland
Schweizerisches Institut für Kinder-und Jugendmedien (SIKJM)	Switzerland
Science and Engineering Research Board (SERB)	India
Science and Technology Department of Zhejiang Province (ZJNSF)	China
Science and Technology Facilities Council (STFC)	United Kingdom
Science Foundation Ireland (SFI)	Ireland
Scientific and Technological Research Council of Turkey (TÜBİTAK)	Turkey
Scleroderma and Raynaud's UK (SRUK)	United Kingdom
Scottish Government	United Kingdom
Sebastian Strong Foundation (SebastianStrongFoundation)	United States
Security Information Service (BIS)	Czechia
Shriners Hospitals for Children (SHC)	United States
Sir Jules Thorn Charitable Trust (Sir JulesThorn CharitableTrust)	United Kingdom
Slovak Research and Development Agency (APVV)	Slovakia
Slovenian Research Agency (ARRS)	Slovenia
Social Sciences and Humanities Research Council (SSHRC)	Canada
Solving Kids' Cancer (SKC)	United States
Southern and Eastern Norway Regional Health Authority	Norway
Sparks (Sparks)	United Kingdom
Spencer Foundation (Spencer)	United States
Spinal Research (Spinal Research)	United Kingdom
St. Baldrick's Foundation (SBF)	United States
State Mining Administration (ČBÚ)	Czechia
State Office for Nuclear Safety (SÚJB)	Czechia
Stroke Association (strokeassociation)	United Kingdom
Substance Abuse and Mental Health Services Administration (SAMHSA)	United States
Susan G. Komen Breast Cancer Foundation (Komen)	United States
Swedish Cancer Society	Sweden
Swedish Energy Agency (Swedish EnergyAgency)	Sweden
Swedish Foundation for Strategic Research (SSF)	Sweden
Swedish Heart-Lung Foundation	Sweden
Swedish Institute (SI)	Sweden

Funding Institution	Host country
Swedish National Space Board (SNSB)	Sweden
Swedish Research Council (SRC)	Sweden
Swedish Research Council for Environment Agricultural Sciences and Spatial Planning (FORMAS)	Sweden
Swedish Research Council for Health Working Life and Welfare (FORTE)	Sweden
Swiss Center for Electronics and Microtechnology (CSEM)	Switzerland
Swiss Federal Institute of Metrology (METAS)	Switzerland
Swiss Federal Nuclear Safety Inspectorate (ENSI)	Switzerland
Swiss Federal Office of Energy (SFOE)	Switzerland
Swiss Institute of Bioinformatics (SIB)	Switzerland
Swiss Institute of Comparative Law (SIR)	Switzerland
Swiss National Science Foundation (SNF)	Switzerland
Swiss Social Archives (SSA)	Switzerland
Swiss Tropical and Public Health Institute (TPH)	Switzerland
Target Ovarian Cancer (Target Ovarian Cancer)	United Kingdom
Team Connor Childhood Cancer Foundation (TeamConnor)	United States
Technology Agency of the Czech Republic (TACR)	Czechia
Telethon Foundation (Telethon)	Italy
Templeton World Charity Foundation (TWCF)	Bahamas
Tenovus Cancer Care (Tenovus)	United Kingdom
Terry Fox Foundation (TFF)	Canada
Thecity of Prague (KHP)	Czechia
The Francis Crick Institute (FCI)	United Kingdom
The Icelandic Centre for Research (RANNIS)	Iceland
The Lewy Body Society (TheLewy Body Society)	United Kingdom
The Lilabean Foundation (LBF)	United States
The Little Princess Trust (TheLittlePrincessTrust)	
The Neuroblastoma Children's Cancer Society (NCCS)	United States
The Neurofibromatosis Therapy Acceleration Program at Johns Hopkins (NTAP)	United States
The Research Council of Norway (RCN)	Norway
The Scar Free Foundation (TheScar FreeFoundation)	United Kingdom
The Steven G. AYA Cancer Research Fund (SGACRF)	United States
The Swifty Foundation (SWIFTY)	United States
The Taylor Matthews Foundation (TAYBANDZ)	United States
The Toyota Foundation	Japan
The Velux Foundations	Denmark
Tobacco-Related Disease Research Program (University of California) (TRDRP)	United States
Tourettes Action (TA)	United Kingdom
Traumatic Brain Injury Center of Excellence (TBICoE)	United States
TSC Alliance (TS Alliance)	United States
Tuberous Sclerosis Association (TSA)	United Kingdom
Ty Louis Campbell Foundation (TLC)	United States
UC Discovery Grants formerly IUCRP (IUCRP)	United States
UC Lab Fees Research Program (UCLRP)	United States
UC Proof of Concept Grant (UCPOC)	United States
UK Research and Innovation (UKRI)	United Kingdom
Uniformed Services University of the Health Sciences (USUHS)	United States

**Funding Institution**	**Host country**
United States Air Force (USAF)	United States
United States Army Corps of Engineers (CoE)	United States
United States Department of Agriculture(USDA)	United States
United States Department of Defense (USDOD)	United States
United States Department of Education (DoED)	United States
United States Department of Energy (DOE)	United States
United States Department of Health and Human Services (DHHS)	United States
United States Department of Homeland Security (DHS)	United States
United States Department of the Air Force (DAF)	United States
United States Department of the Army (DA)	United States
United States Department of the Interior (DOI)	United States
United States Department of the Navy (DON)	United States
United States Department of Transportation (USDOT)	United States
United States Department of Veterans Affairs (VA)	United States
United States Food and Drug Administration (FDA)	United States
United States Geological Survey (USGS)	United States
United States Marine Corps (USMC)	United States
United States National Library of Medicine (NLM)	United States
United States Nuclear Regulatory Commission (NRC)	United States
United States-Israel Binational Science Foundation (BSF)	Israel
University Grants Committee (UGC)	China
University of California-Cancer Research Coordinating Committee (CRCC)	United States
University of California Research Initiatives (UCRI)	United States
University of Malaya (UM)	Malaysia
University of Neuchâtel (SFM)	Switzerland
University of Sharjah (UOS)	United Arab Emirates
Urology Foundation (BUF)	United Kingdom
US Forest Service (USFS)	United States
US DA Rural Development (USDARD)	United States
Ústecký Region (KUL)	Czechia
V Foundation for Cancer Research (V Found)	United States
Versus Arthritis(VA)	United Kingdom
Vienna Science and Technology Fund (WWTF)	Austria
VINNOVA (VINNOVA)	Sweden
Volkswagen Foundation (VolkswagenStiftung)	Germany
W.M.Keck Foundation (W.M.KeckFoundation)	United States
W.K.Kellogg Foundation (WKKF)	United States
Wellbeing of Women (WellbeingofWomen)	United Kingdom
Wellcome Trust (WT)	United Kingdom
Wellcome Trust/DBT India Alliance (IA)	India
Wessex Medical Research (WessexMedical Research)	United Kingdom
Western Norway Regional Health Authority	Norway
William Thomas Grant Foundation (WTGF)	United States
Wine Australia (AGWA)	Australia
Wolfson Foundation (WF)	United Kingdom
Women's Health Initiative (WHI)	United States

Funding Institution	Host country
World Cancer Research Fund International (WCRF)	United Kingdom
World Cancer Research Fund Netherlands (WCRFNL)	Netherlands
World Cancer Research Fund UK (WCRFUK)	United Kingdom
World Health Organization (WHO)	Switzerland
Worldwide Cancer Research (AICR)	United Kingdom
Yorkshire Cancer Research (YCR)	United Kingdom

2. Literature search

We searched PubMed/MEDLINE on 17 October 2022 for publications from 16 Feb 2016 onwards, updating a previously conducted version of this search (EJA Fitchett masters thesis, Harvard University) using the following search terms:

('neonatal[Title/Abstract] OR newborn[Title/Abstract] OR baby[Title/Abstract] OR babies[Title/Abstract] OR infant'[Title/Abstract])) AND ((funding[Title/Abstract] OR funder[Title/Abstract] OR invest[Title/Abstract] OR investment[Title/Abstract] OR finance[Title/Abstract] OR financing[Title/Abstract] OR expenditure[Title/Abstract] OR grant[Title/Abstract] OR award[Title/Abstract] OR spending[Title/Abstract] OR portfolio[Title/Abstract])

n=2355

Titles and abstracts, and if needed full texts, were screened for relevance to the study topic by EL or PA.

3. Search strategy used to search the Dimensions database

We searched the Dimensions database using the following search strategy:

‘newborn* OR neonat* OR baby OR babies OR infant* OR stillbirth* OR stillborn* OR deadborn* OR "dead born" OR "dead borns" OR "fetal death" OR "fetal deaths" OR "fetal demise" OR “foetal death" OR "foetal deaths" OR "foetal demise" OR “fetal mortality" OR "foetal mortality” OR “perinatal death” OR “perinatal deaths” OR “perinatal mortality” OR “intrapartum death” OR “intrapartum deaths” OR “intrapartum demise” OR “intrapartum mortality” OR “intra-partum death” OR “intra-partum deaths” OR “intra-partum demise” OR “intra-partum mortality” OR “intrauterine death” OR “intra-uterine death” OR “intrauterine deaths” OR “intra-uterine deaths” “small vulnerable newborn” OR “small vulnerable newborns” OR “small and vulnerable newborn” OR “small and vulnerable newborns” OR “small sick newborn” OR “small sick newborns” OR “small and sick newborn” OR “small and sick newborns” OR “low birth weight” OR “low birthweight” OR LBW OR “small for gestational age” OR SGA OR “fetal growth restriction” OR FGR OR “intrauterine growth restriction” OR “intra-uterine growth restriction” OR IUGR OR preterm OR pre-term’ in title and abstract;

Active Year is 2020 or 2019 or 2018 or 2017 or 2016 or 2015 or 2014 or 2013 or 2012 or 2011.

4. Table S1. Categorisation of included grants by thematic area of the described research activity

Categorisation of grant thematic areas was adapted from the major causes of neonatal mortality as listed in Oza et.al (2014) (1)

Thematic area	Inclusive of (but not limited to) research related to:
1. Preterm direct complications	- Prevention / understanding aetiology of preterm birth<br>- Surfactant deficiency (Respiratory Distress Syndrome)<br>- Kangaroo mother care<br>- Chronic lung disease / Bronchopulmonary Dysplasia<br>- Intraventricular Haemorrhage<br>- Periventricular Leukomalacia<br>- Necrotizing enterocolitis<br>- Retinopathy of prematurity
2. Intrapartum / birth complications	- Neonatal encephalopathy with criteria suggestive of intrapartum events (including hypoxic ischaemic encephalopathy<br>- Acute intrapartum complications including birth trauma e.g. brachial plexus injury<br>- Newborn resuscitation including delayed cord clamping
3. Neonatal infections	- Sepsis/septicaemia<br>- Meningitis<br>- Pneumonia and other acute respiratory tract infection<br>- Neonatal tetanus<br>- Congenital infections including: malaria, Zika, cytomegalovirus<br>- COVID-19 newborn infection and congenital maternal to newborn infection<br>- Neonatal HIV/ vertical transmission of HIV<br>- Hepatitis B virus (HBV) vertical transmission<br>- Neonatal vaccination (e.g. BCG, HBV)
4. Congenital conditions	- Congenital heart disease<br>- Neural tube defect<br>- Other structural congenital disease<br>- Infantile haemangioma<br>- Genetic conditions e.g. Down syndrome, e.g. ambiguous genitalia e.g. inborn errors of metabolism, e.g. neuromotor diseases<br>- Newborn screening looking to detect congenital conditions
5. Neonatal jaundice	- Physiological or pathological newborn jaundice<br>- Biliary atresia<br>- Haemolytic jaundice<br>- Detection/treatment of neonatal hyperbilirubinemia
6. Stillbirth	- Stillbirth<br>- Foetal death/mortality (that not labelled as miscarriage)<br>- Perinatal and intrapartum deaths
7. Growth restriction / Small for gestational age	- Growth restriction in term babies<br>- Small for gestational age (SGA)/ in-utero growth restriction (IUGR)
8. Neurological conditions and neurodevelopment	- Neonatal stroke<br>- Newborn seizures (not related to HIE)<br>- Neurological physiology, neonatal brain development<br>- Neonatal analgesia and pain<br>- Effects of anaesthetic agents on neonatal brain<br>- Newborn hearing (not newborn hearing screening related),<br>- Newborn sleep,<br>- Early neurodevelopment in neonatal period<br>- Parent-newborn relationship
9. Feeding and nutrition	- Breast-feeding<br>- Formula feeding<br>- Total parenteral nutrition<br>- Breast milk fortification

Thematic area	Inclusive of (but not limited to) research related to:
	- Neonatal nutritional supplements
10. Immunology and the microbiome	- Immunology (newborn immune function, not vaccine or infection related)<br>- Microbiome (including probiotics, not referring to necrotizing enterocolitis)
11. Other sub-specialty conditions and physiology	- Respiratory (e.g. CPAP, e.g. persistent pulmonary hypertension of the newborn (PPHN), lung function)<br>- Metabolic/Endocrine (e.g. metabolic bone disease, thyroid)<br>- Gastrointestinal<br>- Cardiovascular (not congenital heart dis, e.g. normal newborn cardiac physiology)<br>- Renal/urology<br>- Haematology (e.g. blood transfusions, neonatal anaemia)
12. Other specific exposures and outcomes	- Neonatal consequences of maternal lifestyle and/or medication exposure (e.g. smoking, alcohol, opioids, prescribed medication)<br>- Neonatal consequences of (parental) environmental exposure (e.g. pesticides, pollutants, climate change)<br>- Neonatal consequences of COVID-19 maternal exposure or as a result of COVID-19 pandemic (*not* grants looking at (transmission of) infection in the neonate)
13. Non-specific exposure and outcomes	- Non-specific neonatal mortality<br>- Non-defined (e.g. generic term 'birth outcomes') or multiple birth outcomes (e.g. 'preterm delivery, stillbirth, neonatal mortality and growth restriction')<br>- Non-defined (e.g. generic 'improve maternal and newborn health') or multiple 'newborn health' outcomes (e.g. 'birth weight, prematurity, Apgar score, mortality, length of stay and acquisition of nosocomial infections')<br>- Relating generally to 'newborn care' (e.g. infusion pump safety, parental use of medication post discharge)<br>- Postnatal growth/anthropometry/body composition (*not* also referring to nutrition) and gestational age determination (*not* referring to prematurity specifically)

Grants were assigned up to two thematic areas with equal weighting.

5. Table S2. Categorisation of included grants by research pipeline

**Research pipeline category**	**Inclusive of (but not limited to) study types:**
1. Basic science, pre-clinical research, technology development	- Ex-vivo studies<br>- Animal models<br>- Early development of technology/ diagnostic tests/ devices<br>- Funded systematic reviews
2. Observational clinical research, epidemiology	- Ecological studies and studies using population data<br>- Cross-sectional studies<br>- Cohort studies including retrospective or prospective cohort based validations or large clinical cohorts<br>- Clinical studies using imaging/ patient samples (e.g. cord blood, placenta)<br>- Observational studies looking at qualitative or quantitative data on patient experience (unless a programmatic or systems aspect is mentioned)
3. Interventional or experimental research	- Randomised or quasi randomised controlled trials<br>- Pre-post interventional studies
4. Implementation research, complex evaluation, health systems research	- Studies focussed in methods or techniques used to enhance the adoption, implementation, and sustainability of a clinical program or practice<br>- Studies developing/evaluating complex interventions
5. Research-related activities: workforce and infrastructure development, stakeholder engagement	- Funding for research infrastructure<br>- Funding for research workforce development<br>- Development assistance<br>- Funding for community engagement/patient and public involvement and other stakeholder engagement
6. Unspecified research type	Not enough information available in the grant title and/or abstract to define type of study

Grants were assigned up to two pipeline categories with equal weighting.

6. Illustrative examples of included grants and categorisation

(a) Single funder, single recipient

The below example illustrates a grant from a single funding institution to a single research organisation which has a focussed research theme related to 'Preterm direct complications' and lies in the 'Basic science, pre-clinical research, technology development' part of the research pipeline.

grant.9294008

Sex as biological variable in Bronchopulmonary Dysplasia: Role of the Notch pathway

Bronchopulmonary dysplasia (BPD) is a debilitating lung disease with long-term consequences and is one of the most common causes for morbidity in premature neonates. Postnatal exposure to high concentrations of oxygen (hyperoxia) contributes to the development of BPD. Despite the well-established sex-specific differences in the incidence of BPD and impaired lung function in males, the molecular mechanism(s) behind these are not completely understood. Our laboratory has been focused on the study of sex-specific differences in neonatal hyperoxic lung injury. Aberrant Notch signaling contributes to the pathogenesis of many chronic lung diseases and Notch activation is seen in lungs of human infants with BPD. The role of aberrant Notch signaling in pulmonary dysangiogenesis in BPD has not been determined. Critically, neonatal female mice have improved alveolarization and pulmonary vascular development, which is associated with, decreased Notch pathway activation and expression of the Notch ligand Dll4 compared to male littermates in a murine model of BPD. The overall aim of this innovative proposal is to define the role of sex-specific activation of Notch pathway in modulating pulmonary angiogenesis in neonatal hyperoxic lung injury. We hypothesize that decreased Notch activation secondary to lesser Dll4 expression preserves pulmonary angiogenesis in female neonates . The above hypothesis will be tested by the following specific aims: Aim 1: Elucidate the spatio-temporal role of endothelial DLL4 (Notch ligand) in modulating pulmonary angiogenesis. Aim 2: Determine the role pulmonary endothelial Notch signaling in modulating pulmonary angiogenesis in the developing lung exposed to hyperoxia. This proposal will address knowledge gaps in the molecular mechanisms behind the sexual divergent incidence of bronchopulmonary dysplasia and lay the foundation for future sex-specific treatment strategies.

Start date	2020-06-23
End date	2022-05-31
Funding amount (USD)	441039
Country of research organization	United States
Funder name	Eunice Kennedy Shriver National Institute of Child Health and Human Development
Funder group	ICRP - International Cancer Research Partnership; NIH - National Institutes of Health
Research theme (up to two)	Preterm direct complications
Research pipeline (up to two)	Basic science, pre-clinical research, technology development

b) Single funder, multiple recipient

The below example illustrates a grant by a single funding institution to multiple recipient research organisations.
It has a focussed research theme related to 'Neonatal infections' but describes two pipeline elements: 'Basic science, pre-clinical research, technology development' ("to dissect the pathological and immune mechanisms involved", "to characterize [..] genetically the infecting parasites, providing a structural basis for anti-PAM vaccine design") and 'Observational clinical research, epidemiology' (..."the immunological effects will be measured in the…newborns, and in the infant, in relation with timing of infection").).

grant.3780035

Strategies To Prevent Pregnancy-Associated Malaria

The Partners propose to conduct a cohort study in pregnant women and their newborns to quantify the effects of Pregnancy-Associated Malaria (PAM) and to identify a PAM vaccine candidate. Effects of PAM on the pregnant woman (placental infection and anaemia), the offspring (birth weight reduction), and the infant (increased morbidity and mortality) are well known. Studies underlined the role of P. falciparum variable surface antigens expressed on infected erythrocytes in binding to placenta. A specific immune response against this antigen reduces the effect of PAM during latter pregnancies, making possible to develop a new preventive strategy based on the enhancement of this specific response. This goal will be achieved through cohort studies in 2 endemic areas (West and East Africa), as the mechanisms and the resulting effects may vary with transmission. Biological samples will be collected during pregnancy and infancy to dissect the pathological and immune mechanisms involved, as well as to characterize phenotypically and genetically the infecting parasites, providing a structural basis for anti-PAM vaccine design. The immunopathological effects will be measured in the mothers, their newborns, and the infant, in relation with timing of infection. The ultimate goal is to identify the most immunogenic epitopes of VAR2CSA (the major variable surface antigens of P. falciparum parasites infecting the pregnant women) to be included in such a vaccine. It is anticipated that the product of this project will be directly usable to enter in the pipeline of vaccine development. The 7 Partners of the consortium (5 from 4 EU countries, and 2 from Benin and Tanzania) have a combined history of high class, internationally-recognized research in malaria. All EU teams have huge experience of collaboration with malaria endemic countries institutions and with studies related to malaria in pregnant women, that are also routinely conducted by the 2 African Partners.

Start date	2008-02-01
End date	2008-02-01
Funding amount (USD)	4170264
Country of research organization	France; Tanzania; Benin; Netherlands; Denmark; Sweden
Funder name	European Commission
Funder group	-
Research theme (up to two)	Neonatal infections
Research pipeline (up to two)	Basic science, pre-clinical research, technology development;<br>Observational clinical research, epidemiology

c) Implementation research grant with missing funding amount

The below example illustrates a grant by a single funding institution to a single recipient research organisation.
The research theme relates to 'Preterm direct complications' and it is an example of a grant we categorised as 'Implementation research, complex evaluation, health systems research' as it evaluates implementation of an intervention that has an existing evidence base for benefit and is being adapted for implementation in a specific population.
This grant is also illustrative of the 5·1% (345/6,790) of grants in the Objective 2 dataset that did not provide the monetary value of the award and were our direct request to the funders to provide this information (following up over 5 months) was not successful in supplementing this information.
We thus included this grant in any analysis of grant numbers but it did not contribute to analysis of funding amounts.

grant.9231225

Breastfeeding in premature infants: the impact of bfhi for neonatal units

Each year are born premature 15 million worldwide, and Brazil is one of the ten countries with the highest number of premature births, a rate of 9.2% in 2010. Between 2007-2012, the increase in prematurity rate was due mainly to the higher number of cesarean section. Despite advances in neonatal care technologies and subsequent increased survival of these premature infants, prematurity contributes to 45% of deaths among newborns in Brazil. Currently, prematurity and its complications are the leading cause of neonatal mortality and morbidity. The impact in the short and long term of prematurity and its implications is stressful for parents and result in a high cost to the health system in Brazil.
Exclusive breastfeeding (EBF) is an important intervention with low cost to reduce neonatal morbidity and mortality, preventing infections and necrotizing enterocolitis, promoting optimal growth and neurodevelopment and encouraging the mother-baby bond. However, AME rates remain low among premature infants in Brazil, occurring frequently, weaning before discharge. Breastfeeding hospitalized premature is a major challenge due to its physiological and neurological immaturity and difficulty coordinating sucking, swallowing and breathing. Prolonged hospitalization in the neonatal unit can also generate the mother feelings of inadequacy and emotional stress, which can decrease lactation, difficult mother-child bond and delay the initiation of breastfeeding. The systematic and continuous support to mothers by trained professionals is essential to initiate and maintain maternal lactation and maintenance of exclusive breastfeeding until the sixth month of life.
Brazil is internationally recognized for developing initiatives to increase breastfeeding rates and policies have been implemented to promote, protect and support the AME, including the Baby-Friendly Hospital Initiative (BFHI). BFHI is a global strategy that aims to transform the practices, policies and structures of health services to support breastfeeding through implementation of the Ten Steps to Successful Breastfeeding and adoption of the International Code of Substitutes Marketing Breastmilk. BFHI was widely adopted in Brazil and has resulted in significant improvement in support of the initiation and duration of exclusive breastfeeding. However, BFHI was originally designed to support the EBF in newborns healthy term in maternity wards, and does not consider the complex and high technological density of neonatal units context as well as the difficulties in breastfeeding premature.
Thus, despite national and strategies to encourage breastfeeding policies, premature are often deprived of the benefits of exclusive breastfeeding, the promising potential is predictable an innovative intervention that is in BFHI adapted for this population segment risk, called ten Steps to Successful Breastfeeding Exclusive in neonatal units, BFHI-Neo, whose effectiveness will be tested in Brazil with this design proposes its implementation focused on changing practices in Brazilian neonatal units mediated by the knowledge transfer of reference. The goal is to increase the early onset and the prevalence of exclusive breastfeeding; improve the early health conditions related to AME; improve practices to enhance the mother-baby bond and protect, promote and support the EBF in neonatal units. The study objectives are to implement and test the effectiveness of the Three Principles and the Ten Steps to Successful BFHI-Neo Breastfeeding and use PARIHS model (Promoting Action on Research Implementation in Health Services) of the transfer of knowledge and multifaceted and interactive strategy EPIC (Evidence-based practice Identification & change) to promote and support changes in breastfeeding practices in neonatal units in Brazil. Expected to contribute new evidence for the adoption of new national public policies related to breastfeeding in neonatal units.
The ten hospitals / maternity (05 intervention and 05 control) selected to participate in the project are: 1) public or licensed to the Unified Health System, 2) regional reference in care delivery and newborn, 3) with Hospital title Child Friendly or participant's strategy Stork Network of the Ministry of Health, 4) with high rates of prematurity and 5) are located in five regions of the country: north, northeast, Midwest, southeast and south.
The effectiveness of any clinical intervention, including BFHI-Neo, will depend on the implementation and use of intervention by health professionals in practice. The PARIHS model focuses on the relationship between the nature of evidence, the context in which the shift is implemented and the way in which the change is facilitated. The model will guide the use of EPIC, multifaceted intervention knowledge transfer to promote change practices and behavior of health professionals. The successful implementation of BFHI-Neo also requires a strategic plan, with systematic evaluation to monitor the practical improvements and the improvement of care, as well as performed with the original BFHI. Health Practitioners in Quebec, Canada, have developed an electronic program to measure adherence to BFHI originates. These instruments were adapted to measure the adherence to new standards and criteria as the Ten Steps and Three Guiding Principles of BFHI-Neo. These instruments are being tested in a pilot study in Canada and Sweden, and will facilitate data collection, analysis and dissemination of the results of the level of adherence to the BFHI-Neo.

The executor multidisciplinary project team brings together researchers and practitioners with extensive experience in scientific production and care to premature and family and breastfeeding. undergraduate students and graduate students and technicians will be included in the study to provide logistical support. The project includes collaboration with researchers from Canada and Sweden, which developed the BFHI-Neo and their assessment tools, and who also have expertise in knowledge transfer and PARIHS model. The project coordinator has ties and collaboration with strategic professionals and researchers in the participating institutions, which is essential for the successful implementation of BFHI-Neo. The development of research in each region will be the responsibility of a regional coordinator and operational staff. The project monitoring will be done through programs activities in all operational phases of the project, monthly reports, monthly in-person meetings and video conferences, or more frequent, with the participation of the general and regional coordination. Budget execution of the project will be the responsibility of coordinating with the financial support technical assistance from the Ribeirão Preto School of Nursing, University of São Paulo (EERP-USP) and the researchers regional project coordinators.

Start date	2013-12-13
End date	2016-12-31
Funding amount (USD)	not available
Country of research organization	Brazil
Funder name	National Council for Scientific and Technological Development, Brazil
Funder group	-
Research theme (up to two)	Preterm direct complications
Research pipeline (up to two)	Implementation research, complex evaluation, health systems research

7. Table S3. List of funders of included grants

To be included in our analysis and listed here funding organisations had to:

a) be one of >650 funders included in the Dimensions database (dependent on publishing open access data on their research funding activity at an individual grant level)

AND

b) include any of the newborn health or stillbirth related terms included in our search strategy (see above) in the available descriptions/abstracts of their grants

AND

within the pool of all Dimensions grants identified as containing newborn health and stillbirth search terms

c) meet our threshold of being a 'major funder': funding at least 15 of the grants worth ≥$5 million or 75 of the grants worth ≥$1 million (Objective 1 dataset)

OR

fund any grant that names at least one LMIC-hosted recipient institution and was active between 2011-2020 (Objective 2 dataset)

**Funding Institution**	**Host country**
Academy of Medical Sciences	UK
Agricultural Research Service	USA
Alberta Centre for Child, Family and Community Research	Canada
Belgian Federal Science Policy Office	Belgium
Bill & Melinda Gates Foundation	USA
Brain & Behavior Research Foundation	USA
Canadian Institutes of Health Research	Canada
Centers for Disease Control and Prevention	USA
Council for International Exchange of Scholars	USA
Department of Biotechnology	India
Department of Science and Technology	India
Dutch Research Council	Netherlands
Economic and Social Research Council	UK
European & Developing Countries Clinical Trials Partnership	Netherlands
European Union<br>- European Commission<br>- European Research Council	Belgium
Federal Department of Foreign Affairs	Switzerland

**Funding Institution**	**Host country**
Foundation Fighting Blindness	USA
German Research Foundation	Germany
Indian Council of Medical Research	India
Innovation and Technology Commission	China
International Atomic Energy Agency	Austria
International Diabetes Federation	Belgium
Japan Society for the Promotion of Science	Japan
Meningitis Research Foundation	UK
National Council for Scientific and Technological Development	Brazil
National Health and Medical Research Council*	Australia
National Institute for Health Research*	UK
National Institutes of Health<br>- Eunice Kennedy Shriver National Institute of Child Health and Human Development<br>- National Institute on Alcohol Abuse and Alcoholism<br>- National Institute of Mental Health<br>- National Institute of Allergy and Infectious Diseases<br>- Fogarty International Center	USA
National Natural Science Foundation of China	China
National Research Foundation	South Africa
National Science Foundation*	USA
Novo Nordisk Foundation	Denmark
Policy Innovation and Co-ordination Office	China
Qatar National Research Fund	Qatar
Russian Foundation for Basic Research	Russia
Russian Science Foundation	Russia
São Paulo Research Foundation	Brazil
Science and Engineering Research Board	India
Science and Technology Department of Zhejiang Province	China
Swedish Research Council	Sweden
Swiss National Science Foundation	Switzerland
The Research Council of Norway*	Norway
UK Research and Innovation (UKRI)<br>- Engineering and Physical Sciences Research Council<br>- Natural Environment Research Council<br>- Medical Research Council	UK

Funding Institution	Host country
United States Department of Defense*	USA
University Grants Committee	China
Wellcome Trust	UK
Wellcome Trust/DBT India Alliance	India
World Health Organization	Switzerland

* = funder identified as major funder of newborn health and stillbirth research (awarded ≥15 grants worth $5 million or ≥75 grants worth ≥$1 million with newborn health or stillbirth terms in the grant abstract) and featured in the 2019-2020-restricted Objective 1 dataset but did *not* feature in the Objective 2 dataset which was restricted to grants active between 2011-2010 and awarded to at least one LMIC hosted recipient.

8. Table S4. Summary of total new funding awarded per year by major funders (mean, 2019-2020)

	New Funding Awarded per Year (mean, 2019-2020)		Number of New Grants Awarded per Year (mean 2019-2020)		Median Award Size (USD) and Interquartile range (IQR)		Number of grants with missing funding amount
	USD	(%)	n	(%)			
**TOTAL**	**577,139,824**	100.00%	**550**	100.00%	**473,436**	(221,912 - 1,298,209)	21
**Overall thematic group**							
Any grants related to newborn health	574,277,867	(99.5%)	546	(49.6%)	473,436	(222,190 - 1,298,350)	21
Any grants related to small and vulnerable and newborns	166,316,670	(28.8%)	184	(16.7%)	457,112	(264,039 - 1,137,279)	2
Any grants related to stillbirths	14,023,166	(2.4%)	10	(0.9%)	549,452	(112,526 - 2,490,596)	0
Grants specifically related to stillbirth theme	8,442,562	(1.5%)	10	(0.9%)	431,678	(82,886 - 1,245,298)	0
**Specific newborn health research theme**							
Preterm direct complications	131,424,183	(22.8%)	156	(14.1%)	445,504	(244,006 - 1,019,917)	2
Neonatal infections	115,395,352	(20.0%)	79	(7.2%)	472,412	(190,249 - 1,296,542)	1
Congenital conditions	55,469,783	(9.6%)	70	(6.3%)	433,557	(162,250 - 1,183,750)	1
Other specific exposures and outcomes	46,186,219	(8.0%)	53	(4.8%)	671,181	(241,589 - 1,210,195)	4
Neurological conditions and neurodevelopment	33,510,925	(5.8%)	31	(2.8%)	638,815	(137,904 - 1,330,984)	5
Feeding and nutrition	31,562,188	(5.5%)	40	(3.6%)	243,113	(96,165 - 923,711)	1
Other sub-specialty conditions and physiology	22,127,795	(3.8%)	25	(2.3%)	444,241	(207,403 - 1,451,640)	2
Growth restriction / small for gestational age	19,902,779	(3.4%)	34	(3.1%)	404,646	(156,909 - 720,006)	0
Intrapartum/birth complications	18,545,160	(3.2%)	27	(2.5%)	486,247	(208,823 - 1,034,515)	2

Immunology and the microbiome	14,643,840	(2.5%)	17	(1.5%)	433,675	(228,426 - 1,256,903)	1
Neonatal jaundice	3,529,025	(0.6%)	6	(0.5%)	223,488	(34,166 - 1,092,344)	0
Non-specific exposures and outcomes	76,400,017	(13.2%)	69	(6.3%)	485,870	(186,463 - 1,424,753)	2
<u>**Research type/pipeline**</u>							
Observational clinical research, epidemiology	217,828,072	(37.7%)	247	(22.5%)	442,943	(187,224 - 1,110,190)	18
Basic science, pre-clinical research, technology development	173,934,482	(30.1%)	230	(20.9%)	436,061	(220,341 - 1,126,107)	7
Interventional or experimental research	62,687,705	(10.9%)	43	(3.9%)	885,361	(209,298 - 2,091,726)	2
Implementation research, complex evaluations, health systems research	23,241,171	(4.0%)	25	(2.2%)	348,022	(128,416 - 1,306,422)	2
Research-related activity, workforce and infrastructure development, stakeholder engagement	38,353,240	(6.6%)	31	(2.8%)	400,804	(132,929 - 1,993,548)	0
Unspecified research type	61,095,156	(10.6%)	28	(2.5%)	730,955	(313,554 - 1,824,014)	0
<u>**SDG region of recipient organization host country**</u>							
Europe and Northern America	508,986,888	(88.2%)	498	(45.3%)	452,492	(219,517 - 1,306,422)	21
Sub-Saharan Africa	33,048,866	(5.7%)	21	(1.9%)	499,266	(182,477 - 2,070,404)	0
Oceania	27,577,490	(4.8%)	34	(3.0%)	628,287	(389,563 - 1,074,945)	0
Central and Southern Asia	6,449,287	(1.1%)	7	(0.6%)	553,811	(214,586 - 847,530)	0
Northern Africa and Western Asia	732,526	(0.1%)	2	(0.1%)	349,972	(55,455 - 1,059,624)	0
Eastern and South-Eastern Asia	212,005	(0.0%)	1	(0.0%)	424,010	(424,010 - 424,010)	0
Latin America and the Caribbean	132,763	(0.0%)	1	(0.1%)	132,762	(2,232 - 263,292)	1
<u>**World Bank classification of recipient organization host country**</u>							
High-income economies	537,005,079	(93.0%)	530	(48.2%)	466,125	(224,556 - 1,298,350)	21

Upper-middle-income economies	6,070,358	(1.1%)	10	(0.9%)	399,226	(98,781	- 706,416)	0
Lower-middle-income economies	21,029,010	(3.6%)	16	(1.5%)	608,233	(211,942	- 1,451,042)	0
Low-income economies	13,035,377	(2.3%)	6	(0.5%)	1,286,146	(357,530	- 3,178,798)	0
Funding awarded to lower income economy recipients (LMIE/LIE)	34,064,386	(5.9%)	21	(1.9%)	609,719	(214,586	- 2,542,841)	0
Funding awarded to all LMIC recipients (UMIE/LMIE/LIE)	40,134,744	(7.0%)	29	(2.6%)	507,505	(182,477	- 1,993,548)	0

Table S4. Mean annual funding awarded by major funders (2019-2020) - all recipients. Major funders were defined as funders of ≥15 grants worth $5m USD or ≥75 grants worth ≥$1m USD with newborn search terms in the Dimensions database. Funding awarded was assumed to be evenly distributed across categories and recipients, where grants covered multiple categories or awarded to more than one organization. Since up to two thematic and research categories could be coded for each grant, their total proportions can exceed 100%. Sustainable Development Goal (SDG) region country allocations available at: https://unstats.un.org/sdgs/indicators/regional-groups [accessed 5.1.2023]

9. Table S5. All funding received for research related to newborn health or stillbirths via grants with at least one recipient organization in a low- or middle-income country, active between 2011 and 2020

	Funding available from active grants 2011-2020		Number of Active Grants		Median Award Size (USD) and Interquartile range (IQR)		Number of grants with missing funding amount
	USD	(%)	n	(%)			
**TOTAL**	**486,712,214**	(100.0%)	**1,985**	(100.0%)	**37,433**	(23,248 - 87,241)	345
**Overall thematic group**							
Any grants related to newborn health	484,566,629	(99.6%)	1,971	(99.3%)	37,433	(23,248 - 1,630)	341
Any grants related to small and vulnerable and newborns	73,136,878	(15.0%)	517	(26.0%)	32,772	(21,364 - 444)	73
Any grants related to stillbirths	21,864,954	(4.5%)	30	(1.5%)	92,573	(25,576 - 26)	4
Grants specifically related to stillbirth theme	12,005,270	(2.5%)	30	(1.5%)	49,833	(12,788 - 332,122)	4
**Specific newborn health research theme**							
Neonatal infections	201,954,256	(41.5%)	361	(18.2%)	49,693	(22,479 - 244,239)	90
Preterm direct complications	44,438,962	(9.1%)	386	(19.4%)	31,667	(14,667 - 74,802)	63
Congenital conditions	28,593,361	(5.9%)	299	(15.1%)	40,950	(23,109 - 85,204)	32
Other sub-specialty conditions and physiology	20,817,688	(4.3%)	102	(5.1%)	37,866	(21,579 - 89,044)	12
Feeding and nutrition	16,224,987	(3.3%)	177	(8.9%)	30,680	(12,788 - 76,589)	57
Growth restriction / small for gestational age	16,196,589	(3.3%)	153	(7.7%)	32,818	(19,901 - 91,110)	20
Other specific exposures and outcomes	10,068,250	(2.1%)	90	(4.5%)	35,916	(16,693 - 71,154)	16
Intrapartum/birth complications	8,476,452	(1.7%)	183	(9.2%)	33,386	(25,576 - 72,607)	20
Neurological conditions and neurodevelopment	6,503,378	(1.3%)	138	(7.0%)	33,818	(18,881 - 59,444)	25
Immunology and the microbiome	2,573,098	(0.5%)	44	(2.2%)	33,060	(10,682 - 57,120)	1

Neonatal jaundice	1,878,070	(0.4%)	52	(2.6%)	30,877	(22,364	-	40,950)	3
Non-specific exposures and outcomes	116,981,853	(24.0%)	149	(7.5%)	81,268	(11,095	-	721,703)	35
**Research type/pipeline**									
Observational clinical research, epidemiology	158,004,037	(32.5%)	889	(44.8%)	29,674	(6,722	-	78,711)	221
Basic science, pre-clinical research, technology development	78,804,046	(16.2%)	1,034	(52.1%)	33,589	(24,352	-	72,656)	87
Interventional or experimental research	99,272,118	(20.4%)	97	(4.9%)	360,526	(53,398	-	1,503,656)	16
Implementation research, complex evaluations, health systems research	13,888,372	(2.9%)	39	(2.0%)	89,535	(353,123	-	353,123)	9
Research-related activity, workforce and infrastructure development, stakeholder engagement	49,816,725	(10.2%)	36	(1.8%)	677,125	(34,000	-	1,543,928)	2
Unspecified research type	86,926,916	(17.9%)	97	(4.9%)	77,535	(27,375	-	441,000)	23
**SDG region of recipient organization host country**									
Europe and Northern America	155,122,244	(31.9%)	188	(9.5%)	562,951	(140,629	-	1,481,932)	80
Sub-Saharan Africa	146,706,762	(30.1%)	237	(11.9%)	267,474	(87,742	-	947,073)	81
Central and Southern Asia	93,718,685	(19.3%)	118	(5.9%)	103,514	(39,792	-	343,609)	16
Eastern and South-Eastern Asia	58,745,325	(12.1%)	958	(48.3%)	35,841	(28,007	-	81,900)	9
Latin America and the Caribbean	19,952,173	(4.1%)	603	(30.4%)	10,676	(2,361	-	42,469)	173
Oceania	8,315,928	(1.7%)	18	(0.9%)	246,114	(90,190	-	737,594)	4
Northern Africa and Western Asia	5,576,040	(1.1%)	17	(0.9%)	153,328	(55,625	-	374,591)	4

Table S5. Funding awarded was assumed to be evenly distributed across categories and recipients, where grants covered multiple categories or awarded to more than one organization. Since up to two thematic and research categories could be coded for each grant, their total proportions can exceed 100%. Active funding takes into account all available funding per year, under the assumption that grants are evenly dispersed across their duration, with totals apportioned to that available between 2011 and 2020.

10. Figure S1. Heatmaps of funding (USD) awarded in 2019 and 2020 from major funders, by research theme and research pipeline categories across SDG super region

	Europe and Northern America	Sub-Saharan Africa	Oceania	Central and Southern Asia	Northern Africa and Western Asia	Eastern and South-Eastern Asia	Latin America and the Caribbean
**Research type/pipeline**							
Observational clinical research, epidemiology	400,507	15,479	16,616	2,350	705	0	0
Basic science, preclinical research, technology development	331,327	1,316	12,304	2,659	0	0	263
Interventional/experimental research	95,536	19,258	7,078	3,080	0	424	0
Unspecified research type	94,916	8,851	13,613	4,810	0	0	0
Research-related activity	65,017	9,079	1,847	0	760	0	2
Implementation/complex evaluation/health systems research	30,672	12,115	3,696	0	0	0	0
**Thematic area**							
Preterm direct complications	232,359	6,182	19,462	4,846	0	0	0
Neonatal infections	191,637	33,275	2,614	3,209	55	0	0
Non-specific exposures and outcomes	116,161	18,628	13,613	4,398	0	0	0
Congenital conditions	105,045	350	3,976	0	0	0	0
Other specific exposures and outcomes	89,549	486	1,708	100	530	0	0
Neurological conditions and neurodevelopment	66,251	200	571	0	0	0	0
Feeding and nutrition	60,482	281	1,405	0	530	424	2
Other subspecialty conditions and physiology	43,347	304	605	0	0	0	0
Growth restriction/small for gestational	35,869	244	3,429	0	0	0	263
Intrapartum/birth complications	35,267	0	1,823	0	0	0	0
Immunology and the microbiome	29,288	0	0	0	0	0	0
Stillbirth	5,661	4,930	5,949	345	0	0	0
Neonatal jaundice	7,058	0	0	0	0	0	0

Figure S1. Funding shown represents the total value of new grants awarded in 2019 and 2020 by 'major funders' of newborn health or stillbirth research as allocated Sustainable Development Goal (SDG) region by research pipeline category or by grant thematic area.[2] Awards were assumed to be evenly distributed across categories whe grants covered multiple categories or were awarded to more than one organisation.

11.Table S6. Total funding (USD) active between 2011-2020 awarded to at least one LMIC recipient, by year and by thematic area, pipeline, funders and recipients

	2011		2012		2013		2014		2015	
	n	USD (%)	n	USD (%)	n	USD (%)	n	USD (%)	n	USD (%)
**TOTAL**	295	23,501,934 (100.0%)	361	27,696,168 (100.0%)	486	31,819,154 (100.0%)	514	40,507,876 (100.0%)	585	52,365,150 (100.0%)
**Overall thematic group**										
Any grants related to newborn health	294	23,501,934 (100.0%)	360	27,654,149 (99.8%)	482	31,763,177 (99.8%)	513	40,450,991 (99.9%)	584	52,351,575 (100.0%)
Any grants related to small and vulnerable and newborns	69	2,720,533 (11.6%)	90	3,326,665 (12.0%)	130	4,501,959 (14.1%)	142	6,123,575 (15.1%)	150	7,427,371 (14.2%)
Any grants related to stillbirths	5	1,248,272 (5.3%)	6	1,437,525 (5.2%)	9	1,477,296 (4.6%)	5	1,491,448 (3.7%)	5	1,501,155 (2.9%)
**Distribution of grants across individual thematic areas**										
Non-specific exposures and outcomes	28	8,792,902 (37.4%)	33	10,383,136 (37.5%)	41	8,312,205 (26.1%)	39	10,552,883 (26.1%)	44	11,231,145 (21.4%)
Preterm direct complications	53	1,931,879 (8.2%)	66	2,244,903 (8.1%)	95	2,645,968 (8.3%)	103	4,045,646 (10.0%)	113	5,058,975 (9.7%)
Intrapartum/birth complications	28	374,141 (1.6%)	46	625,496 (2.3%)	52	767,410 (2.4%)	50	828,724 (2.0%)	55	866,350 (1.7%)
Neonatal infections	47	6,771,882 (28.8%)	50	7,469,831 (27.0%)	68	10,146,987 (31.9%)	71	13,317,968 (32.9%)	86	23,017,183 (44.0%)
Congenital conditions	49	1,381,082 (5.9%)	65	2,380,329 (8.6%)	81	3,007,594 (9.5%)	85	3,144,955 (7.8%)	105	3,640,212 (7.0%)
Neonatal jaundice	8	71,355 (0.3%)	3	26,967 (0.1%)	9	107,157 (0.3%)	11	137,549 (0.3%)	17	240,253 (0.5%)
Stillbirth	5	624,136 (2.7%)	6	739,772 (2.7%)	9	766,636 (2.4%)	5	774,166 (1.9%)	5	757,365 (1.4%)
Growth restriction / small for gestational	17	239,122 (1.0%)	25	553,897 (2.0%)	40	1,031,677 (3.2%)	42	1,214,222 (3.0%)	43	1,468,700 (2.8%)
Neurological conditions and neurodevelopment	23	1,743,063 (7.4%)	28	454,830 (1.6%)	30	406,342 (1.3%)	30	300,223 (0.7%)	31	398,812 (0.8%)
Feeding and nutrition	21	268,466 (1.1%)	26	474,383 (1.7%)	40	1,451,036 (4.6%)	48	1,875,227 (4.6%)	52	1,259,157 (2.4%)
Immunology and the microbiome	8	120,772 (0.5%)	8	77,048 (0.3%)	11	220,760 (0.7%)	13	330,050 (0.8%)	13	316,307 (0.6%)
Other sub-specialty conditions and physiology	24	622,144 (2.6%)	23	1,692,101 (6.1%)	35	2,276,929 (7.2%)	32	3,307,105 (8.2%)	33	3,327,908 (6.4%)
Other specific exposures and outcomes	10	560,990 (2.4%)	12	573,477 (2.1%)	20	678,453 (2.1%)	22	679,156 (1.7%)	30	782,784 (1.5%)
**Research type/pipeline**										
Basic science	160	4,726,426 (20.1%)	194	5,330,190 (19.2%)	255	7,812,354 (24.6%)	276	8,619,926 (21.3%)	327	9,397,942 (17.9%)
Observational/epidemiology	131	5,909,229 (25.1%)	161	6,522,207 (23.5%)	212	7,952,781 (25.0%)	220	11,585,636 (28.6%)	238	18,327,430 (35.0%)
Interventional/experimental	14	2,268,858 (9.7%)	18	3,451,737 (12.5%)	29	5,703,379 (17.9%)	39	6,894,475 (17.0%)	47	10,111,285 (19.3%)
Implementation/complex evaluation/health systems research	5	1,642,132 (7.0%)	9	1,757,759 (6.3%)	14	1,905,373 (6.0%)	14	1,468,103 (3.6%)	13	586,792 (1.1%)
Research-related activity	9	1,927,690 (8.2%)	9	3,183,211 (11.5%)	10	3,977,348 (12.5%)	14	3,889,932 (9.6%)	12	4,161,102 (7.9%)
Unspecified	11	7,027,598 (29.9%)	11	7,451,065 (26.9%)	21	4,467,919 (14.0%)	13	8,049,804 (19.9%)	17	9,780,599 (18.7%)
**Funding given, by SDG Super region**										
Sub-Saharan Africa	0	0 (0.0%)	0	0 (0.0%)	0	0 (0.0%)	0	0 (0.0%)	0	0 (0.0%)
Northern Africa and Western Asia	0	0 (0.0%)	0	0 (0.0%)	0	0 (0.0%)	0	0 (0.0%)	0	0 (0.0%)
Central and Southern Asia	9	119,617 (0.5%)	13	270,138 (1.0%)	13	270,138 (0.8%)	16	1,305,226 (3.2%)	17	1,344,406 (2.6%)
Eastern and South-Eastern Asia	140	3,276,952 (13.9%)	171	3,267,925 (11.8%)	235	4,621,413 (14.5%)	244	5,350,657 (13.2%)	309	6,666,331 (12.7%)
Latin America and the Caribbean	74	1,195,628 (5.1%)	106	1,619,655 (5.8%)	137	1,817,418 (5.7%)	168	1,551,737 (3.8%)	161	1,189,864 (2.3%)
Oceania	0	0 (0.0%)	0	0 (0.0%)	0	0 (0.0%)	0	0 (0.0%)	0	0 (0.0%)
Europe and Northern America	52	18,909,737 (80.5%)	57	22,538,450 (81.4%)	69	25,010,458 (78.6%)	82	32,300,256 (79.7%)	96	43,164,548 (82.4%)
**Funding received*, by SDG Super region**										
Sub-Saharan Africa	49	5,165,399 (22.0%)	49	7,647,885 (27.6%)	73	10,170,122 (32.0%)	51	13,647,753 (33.7%)	57	16,647,263 (31.8%)
Northern Africa and Western Asia	5	43,353 (0.2%)	5	96,152 (0.3%)	6	170,861 (0.5%)	8	210,527 (0.5%)	7	586,648 (1.1%)
Central and Southern Asia	20	6,466,672 (27.5%)	20	6,819,854 (24.6%)	24	3,569,332 (11.2%)	33	6,560,301 (16.2%)	39	10,257,157 (19.6%)
Eastern and South-Eastern Asia	147	3,416,570 (14.5%)	178	3,470,721 (12.5%)	244	4,933,030 (15.5%)	252	5,606,009 (13.8%)	319	6,911,569 (13.2%)
Latin America and the Caribbean	80	1,321,156 (5.6%)	110	1,687,554 (6.1%)	141	1,836,240 (5.8%)	173	1,839,335 (4.5%)	165	1,792,099 (3.4%)
Oceania	5	44,736 (0.2%)	5	193,816 (0.7%)	8	413,719 (1.3%)	7	462,669 (1.1%)	8	412,700 (0.8%)
Europe and Northern America	31	7,044,047 (30.0%)	33	7,780,186 (28.1%)	39	10,725,851 (33.7%)	43	12,181,281 (30.1%)	46	15,757,713 (30.1%)
**Funding given, by funder host country**										
**World Bank Classificaton**										
High-income economies	50	18,909,737 (80.5%)	52	22,538,450 (81.4%)	66	25,010,458 (78.6%)	79	32,307,855 (79.8%)	95	43,184,442 (82.5%)
Upper-middle-income economies	236	4,472,580 (19.0%)	296	4,887,580 (17.6%)	407	6,438,830 (20.2%)	419	6,894,795 (17.0%)	473	7,836,302 (15.0%)
Lower-middle-income economies	9	119,617 (0.5%)	13	270,138 (1.0%)	13	369,866 (1.2%)	16	1,305,226 (3.2%)	17	1,344,406 (2.6%)
Low-income economies	0	0 (0.0%)	0	0 (0.0%)	0	0 (0.0%)	0	0 (0.0%)	0	0 (0.0%)
Funding given by all LMIC funders **(UMIE/LMIE/LIE)**	245	4,592,197 (19.5%)	309	5,157,718 (18.6%)	420	6,808,696 (21.4%)	435	8,200,021 (20.2%)	490	9,180,707 (17.5%)
**Funding received*, by recipient organization host country**										
**World Bank Classificaton**										
High-income economies	29	7,016,461 (29.9%)	28	7,929,365 (28.6%)	35	11,082,788 (34.8%)	38	12,485,440 (30.8%)	44	16,095,111 (30.7%)
Upper-middle-income economies	258	6,072,309 (25.8%)	319	6,800,772 (24.6%)	435	8,973,429 (28.2%)	452	10,772,523 (26.6%)	512	14,208,776 (27.1%)
Lower-middle-income economies	37	8,323,958 (35.4%)	40	10,729,040 (38.7%)	48	9,225,294 (29.0%)	60	14,800,447 (36.5%)	66	19,552,660 (37.3%)
Low-income economies	14	2,089,205 (8.9%)	15	2,236,991 (8.1%)	17	2,537,643 (8.0%)	17	2,449,465 (6.0%)	20	2,508,602 (4.8%)
Funding awarded to all LMIC recipients **(UMIE/LMIE/LIE)**	295	16,485,472 (70.1%)	361	19,766,803 (71.4%)	486	20,736,366 (65.2%)	514	28,022,436 (69.2%)	585	36,270,038 (69.3%)

	2016		2017		2018		2019		2,020	
	n	USD (%)	n	USD (%)	n	USD (%)	n	USD (%)	n	USD (%)
**TOTAL**	637	56,272,603 (100.0%)	661	60,290,758 (100.0%)	705	60,501,287 (100.0%)	654	65,511,751 (100.0%)	579	68,245,538 (100.0%)
**Overall thematic group**										
Any grants related to newborn health	637	56,272,603 (100.0%)	659	60,215,965 (99.9%)	698	59,920,459 (99.0%)	647	64,874,503 (99.0%)	571	67,561,277 (99.0%)
Any grants related to small and vulnerable and newborns	160	8,572,687 (15.2%)	163	8,841,893 (14.7%)	184	10,287,848 (17.0%)	177	10,840,143 (16.5%)	154	10,494,205 (15.4%)
Any grants related to stillbirths	6	1,689,357 (3.0%)	8	2,039,662 (3.4%)	11	2,926,589 (4.8%)	11	3,099,429 (4.7%)	14	4,954,222 (7.3%)
**Distribution of grants across individual thematic areas**										
Non-specific exposures and outcomes	49	12,990,095 (23.1%)	41	15,424,230 (25.6%)	39	12,830,224 (21.2%)	43	12,397,686 (18.9%)	44	14,067,347 (20.6%)
Preterm direct complications	122	5,644,908 (10.0%)	112	5,837,857 (9.7%)	129	5,823,234 (9.6%)	128	5,740,942 (8.8%)	114	5,464,652 (8.0%)
Intrapartum/birth complications	54	865,751 (1.5%)	50	912,281 (1.5%)	56	1,204,554 (2.0%)	53	1,068,744 (1.6%)	50	963,000 (1.4%)
Neonatal infections	118	24,896,177 (44.2%)	144	25,773,082 (42.7%)	156	26,506,532 (43.8%)	151	31,361,492 (47.9%)	138	32,693,124 (47.9%)
Congenital conditions	111	3,215,998 (5.7%)	104	3,203,731 (5.3%)	104	2,977,049 (4.9%)	76	2,934,982 (4.5%)	60	2,707,430 (4.0%)
Neonatal jaundice	20	253,175 (0.4%)	19	247,836 (0.4%)	23	325,785 (0.5%)	19	242,274 (0.4%)	13	225,719 (0.3%)
Stillbirth	6	844,679 (1.5%)	8	1,057,227 (1.8%)	11	1,753,708 (2.9%)	11	1,868,338 (2.9%)	14	2,819,241 (4.1%)
Growth restriction / small for gestational	46	1,639,722 (2.9%)	59	1,887,063 (3.1%)	66	2,650,061 (4.4%)	58	2,932,963 (4.5%)	46	2,579,162 (3.8%)
Neurological conditions and neurodevelopment	42	553,796 (1.0%)	47	690,439 (1.1%)	50	830,515 (1.4%)	46	655,811 (1.0%)	41	469,547 (0.7%)
Feeding and nutrition	53	976,881 (1.7%)	67	1,150,388 (1.9%)	62	2,263,292 (3.7%)	59	3,584,327 (5.5%)	51	2,921,830 (4.3%)
Immunology and the microbiome	11	346,491 (0.6%)	12	351,268 (0.6%)	14	346,496 (0.6%)	11	257,587 (0.4%)	12	206,320 (0.3%)
Other sub-specialty conditions and physiology	37	3,185,262 (5.7%)	37	2,979,992 (4.9%)	33	2,389,846 (4.0%)	35	536,701 (0.8%)	25	499,701 (0.7%)
Other specific exposures and outcomes	31	859,669 (1.5%)	28	775,363 (1.3%)	26	599,990 (1.0%)	29	1,929,903 (2.9%)	32	2,628,465 (3.9%)
**Research type/pipeline**										
Basic science	336	9,579,917 (17.0%)	352	8,760,670 (14.5%)	408	9,960,036 (16.5%)	337	7,885,968 (12.0%)	272	6,730,616 (9.9%)
Observational/epidemiology	276	19,445,393 (34.6%)	310	22,489,045 (37.3%)	291	22,110,663 (36.5%)	281	22,354,405 (34.1%)	265	21,307,248 (31.2%)
Interventional/experimental	50	10,850,586 (19.3%)	37	11,039,275 (18.3%)	34	12,709,964 (21.0%)	40	16,899,427 (25.8%)	38	19,343,133 (28.3%)
Implementation/complex evaluation/health systems research	14	544,041 (1.0%)	11	333,023 (0.6%)	11	355,538 (0.6%)	12	1,120,427 (1.7%)	13	4,175,185 (6.1%)
Research-related activity	14	3,838,280 (6.8%)	12	5,866,529 (9.7%)	14	6,082,139 (10.1%)	13	8,567,585 (13.1%)	13	8,322,909 (12.2%)
Unspecified	22	12,014,384 (21.4%)	23	11,802,217 (19.6%)	31	9,282,947 (15.3%)	43	8,683,939 (13.3%)	40	8,366,447 (12.3%)
**Funding given, by SDG Super region**										
Sub-Saharan Africa	0	0 (0.0%)	0	0 (0.0%)	0	0 (0.0%)	0	0 (0.0%)	0	0 (0.0%)
Northern Africa and Western Asia	0	0 (0.0%)	0	0 (0.0%)	0	0 (0.0%)	0	0 (0.0%)	0	0 (0.0%)
Central and Southern Asia	16	1,431,805 (2.5%)	15	1,484,303 (2.5%)	26	1,836,502 (3.0%)	26	1,729,038 (2.6%)	17	1,539,489 (2.3%)
Eastern and South-Eastern Asia	322	6,741,984 (12.0%)	346	6,704,981 (11.1%)	405	8,281,036 (13.7%)	314	6,137,837 (9.4%)	254	5,350,730 (7.8%)
Latin America and the Caribbean	182	896,820 (1.6%)	191	989,793 (1.6%)	154	1,047,607 (1.7%)	189	1,333,628 (2.0%)	186	1,213,083 (1.8%)
Oceania	0	0 (0.0%)	0	0 (0.0%)	0	0 (0.0%)	0	0 (0.0%)	0	0 (0.0%)
Europe and Northern America	115	47,201,993 (83.9%)	105	51,111,681 (84.8%)	120	49,336,142 (81.5%)	125	56,311,249 (86.0%)	122	60,142,236 (88.1%)
**Funding received*, by SDG Super region**										
Sub-Saharan Africa	61	16,824,538 (29.9%)	62	17,485,566 (29.0%)	69	15,728,310 (26.0%)	70	19,010,682 (29.0%)	73	24,379,244 (35.7%)
Northern Africa and Western Asia	5	599,647 (1.1%)	3	962,644 (1.6%)	5	1,075,295 (1.8%)	7	1,381,193 (2.1%)	6	449,720 (0.7%)
Central and Southern Asia	43	11,946,025 (21.2%)	38	11,251,977 (18.7%)	48	12,789,111 (21.1%)	52	13,382,583 (20.4%)	45	10,675,675 (15.6%)
Eastern and South-Eastern Asia	334	7,009,310 (12.5%)	353	7,098,669 (11.8%)	410	8,556,458 (14.1%)	319	6,334,770 (9.7%)	257	5,408,218 (7.9%)
Latin America and the Caribbean	191	1,756,964 (3.1%)	200	2,499,094 (4.1%)	160	2,566,557 (4.2%)	194	2,382,085 (3.6%)	191	2,271,090 (3.3%)
Oceania	10	1,248,122 (2.2%)	10	1,489,465 (2.5%)	8	1,421,781 (2.4%)	7	1,407,219 (2.1%)	5	1,221,701 (1.8%)
Europe and Northern America	58	16,887,997 (30.0%)	50	19,503,343 (32.3%)	56	18,363,774 (30.4%)	52	21,613,219 (33.0%)	48	23,839,891 (34.9%)
**Funding given, by funder host country**										
**World Bank Classificaton**										
High-income economies	108	47,225,449 (83.9%)	98	51,135,570 (84.8%)	107	49,342,110 (81.6%)	111	56,311,249 (86.0%)	111	60,142,236 (88.1%)
Upper-middle-income economies	513	7,615,349 (13.5%)	548	7,670,885 (12.7%)	572	9,322,675 (15.4%)	517	7,471,464 (11.4%)	451	6,563,813 (9.6%)
Lower-middle-income economies	16	1,431,805 (2.5%)	15	1,484,303 (2.5%)	26	1,836,502 (3.0%)	26	1,729,038 (2.6%)	17	1,539,489 (2.3%)
Low-income economies	0	0 (0.0%)	0	0 (0.0%)	0	0 (0.0%)	0	0 (0.0%)	0	0 (0.0%)
Funding given by all LMIC funders **(UMIE/LMIE/LIE)**	529	9,047,154 (16.1%)	563	9,155,188 (15.2%)	598	11,159,177 (18.4%)	543	9,200,502 (14.0%)	468	8,103,302 (11.9%)
**Funding received*, by recipient organization host country**										
**World Bank Classificaton**										
High-income economies	50	17,206,962 (30.6%)	42	20,016,420 (33.2%)	42	18,801,869 (31.1%)	38	22,305,194 (34.0%)	37	24,356,557 (35.7%)
Upper-middle-income economies	554	14,445,526 (25.7%)	587	15,299,477 (25.4%)	615	17,608,626 (29.1%)	559	16,881,189 (25.8%)	493	16,797,439 (24.6%)
Lower-middle-income economies	76	21,718,193 (38.6%)	70	21,422,021 (35.5%)	87	20,859,624 (34.5%)	91	21,227,281 (32.4%)	80	19,414,651 (28.4%)
Low-income economies	23	2,901,922 (5.2%)	22	3,552,839 (5.9%)	23	3,231,167 (5.3%)	22	5,098,087 (7.8%)	23	7,676,891 (11.2%)
Funding awarded to all LMIC recipients **(UMIE/LMIE/LIE)**	637	39,065,641 (69.4%)	661	40,274,338 (66.8%)	705	41,699,417 (68.9%)	654	43,206,557 (66.0%)	579	43,888,981 (64.3%)

*Funding assumed to be spread evenly across multiple recipient organizations

12. Table S7. Summary of mean total new funding awarded per year by all funders in grants with at least one LMIC-hosted recipient organization (mean 2019-2020)

	Total new funding awarded per year (mean, 2019-2020)		Mean number of new grants awarded per year		Median award size (USD) and interquartile range (IQR)		
	USD	(%)	n	(%)			
**TOTAL**	**74,311,914**	100.00%	**267**	100.00%	**70,368**	(30,185-	136,911)
**Overall thematic group**							
Any grants related to newborn health	74,247,701	(99.9%)	133	(49.6%)	70,368	(30,185-	142,057)
Any grants related to small and vulnerable and newborns	9,000,010	(12.1%)	41	(15.4%)	70,368	(30,643-	85,596)
Any grants related to stillbirths	6,266,969	(8.4%)	4	(1.3%)	126,291	(37,000-	4,830,943)
Grants specifically related to stillbirth theme	3,165,591	(4.3%)	4	(1.3%)	64,208	(18,500-	2,415,472)
**Specific newborn health research theme**							
Preterm direct complications	6,139,314	(8.3%)	34	(12.7%)	57,855	(30,414 -	86,812)
Neonatal infections	34,631,436	(46.6%)	29	(10.7%)	91,443	(16,780 -	609,719)
Congenital conditions	2,225,446	(3.0%)	12	(4.3%)	31,170	(28,257 -	84,104)
Other specific exposures and outcomes	5,150,681	(6.9%)	10	(3.6%)	85,578	(3,389 -	147,204)
Neurological conditions and neurodevelopment	161,819	(0.2%)	8	(3.0%)	25,403	(1,973 -	33,762)
Feeding and nutrition	4,885,537	(6.6%)	12	(4.3%)	30,643	(2,213 -	104,649)

Other sub-specialty conditions and physiology	321,465	(0.4%)	6	(2.1%)	41,736	(2,268 -	235,610)
Growth restriction / small for gestational age	759,286	(1.0%)	9	(3.2%)	65,336	(30,185 -	84,104)
Intrapartum/birth complications	573,373	(0.8%)	10	(3.7%)	83,472	(30,643 -	84,104)
Immunology and the microbiome	83,518	(0.1%)	3	(1.1%)	19,361	(1,367 -	41,736)
Neonatal jaundice	170,823	(0.2%)	3	(0.9%)	76,688	(61,398 -	78,200)
Non-specific exposures and outcomes	16,043,630	(21.6%)	13	(4.7%)	214,586	(2,268 -	3,508,604)
**<u>Research type/pipeline</u>**							
Observational clinical research, epidemiology	21,868,627	(29.4%)	67	(25.1%)	37,000	(2,213 -	136,911)
Basic science, pre-clinical research, technology development	4,037,658	(5.4%)	51	(18.9%)	38,396	(30,185 -	84,104)
Interventional or experimental research	23,423,595	(31.5%)	10	(3.6%)	2,575,074	(182,626 -	5,012,371)
Implementation research, complex evaluations, health systems research	6,915,449	(9.3%)	4	(1.5%)	530,312	(110,367 -	2,809,639)
Research-related activity, workforce and infrastructure development, stakeholder engagement	11,457,214	(15.4%)	4	(1.5%)	2,200,257	(764,882 -	3,836,789)
Unspecified research type	6,609,372	(8.9%)	13	(4.7%)	82,630	(30,643 -	98,761)
**<u>SDG region of recipient organization host country</u>**							
Europe and Northern America	32,262,112	(43.4%)	19	(6.9%)	2,288,295	(463,000 -	4,951,493)
Sub-Saharan Africa	31,191,873	(42.0%)	20	(7.5%)	463,000	(169,135 -	2,035,146)
Oceania	0	(0.0%)	0	(0.0%)	-	-	
Central and Southern Asia	6,525,144	(8.8%)	10	(3.7%)	507,505	(128,416 -	725,000)

Northern Africa and Western Asia	732,526	(1.0%)	2	(0.6%)	349,972	(55,455 -	1,059,624)
Eastern and South-Eastern Asia	2,756,722	(3.7%)	41	(15.4%)	81,194	(30,643 -	84,104)
Latin America and the Caribbean	843,537	(1.1%)	53	(19.7%)	2,240	(2,085 -	37,000)
**World Bank classification of recipient organization host country**							
High-income economies	32,702,814	(44.0%)	8	(3.0%)	2,288,295	(463,000 -	4,951,493)
Upper-middle-income economies	9,537,855	(12.8%)	112	(41.9%)	37,969	(13,635 -	84,104)
Lower-middle-income economies	19,100,077	(25.7%)	19	(7.1%)	447,766	(136,911 -	1,253,093)
Low-income economies	12,971,169	(17.5%)	6	(2.1%)	1,319,200	(445,040 -	3,898,449)

Table S7: Funding amounts and numbers of grants presented for each category are the mean of those newly awarded by all funders in 2019 or 2020. In total, 163 new grants worth $82,495,313 and 104 new grants worth $66,128,514 with at least one LMIC-hosted recipient organization were awarded by all funders in 2019 and 2020 respectively. Median award sizes for each category are based on the portion of awards available between 2011 and 2020 only. Grants may be relevant to more than one overall thematic category (and therefore the sum of proportions for overall thematic categories may exceed 100%). Otherwise, where grants were related to multiple theme or pipeline categories, or were awarded to more than one organization, they were assumed to be evenly apportioned. The funding amount was missing for 69 grants. Countries of recipients were categorised according to Sustainable Development Goal (SDG) region (2) and World Bank economic status (3)

13.Table S8. Total funding (USD) active between 2011-2020 awarded to at least one LMIC recipient, number of active grants and median available funding per grant (USD) and interquartile range (IQR), by recipients and funder

	Total Active Funding*		Number of Active Grants*		Median Available Funding per Grant (USD) and Interquartile range (IQR)			Number of grants with missing funding data
	USD (%)		n (%)					
**TOTAL**	**486,712,214**	100.0%	**1,985**	100.0%	**37,433**	(23,248 -	87,241)	345
**Funding given, by funder host country World Bank Classification**								
High-income economies	406,107,554	83.4%	277	14.0%	417,097	(98,761 -	1,562,962)	35
Upper-middle-income economies	69,174,272	14.2%	1,654	83.3%	33,314	(20,160 -	71,154)	305
Lower-middle-income economies	11,430,389	2.3%	54	2.7%	68,881	(32,185 -	109,611)	5
Low-income economies	0	0.0%	0	0.0%	0	(0 -	0)	-
Funding given by all LMIC funders	80,604,661	16.6%	1,762	88.8%	33,386	(21,163 -	71,555)	364
**Funding received, by recipient organization host country World Bank Classification**								
High-income economies	157,296,168	32.3%	108	5.4%	661,511	(178,613 -	1,618,847)	5
Upper-middle-income economies	127,860,066	26.3%	1,759	88.6%	33,818	(21,163 -	77,269)	317
Lower-middle-income economies	167,273,168	34.4%	203	10.2%	155,602	(61,980 -	507,469)	23
Low-income economies	34,282,814	7.0%	62	3.1%	384940	(89,701 -	808,828)	9
Funding awarded to all LMIC recipients	329,416,048	67.7%	1,985	100.0%	36,670	(22,763 -	85,999)	345

**<u>Top 10 funding organizations or funder groups (by total active funding 2011-2020)</u>**								
Bill & Melinda Gates Foundation (USA)	117,116,089	24.1%	59	3.0%	355,314	(92,156 -	1,503,349)	0
European Union (European Commission/European Research Council) (EU)	97,461,227	20.0%	27	1.4%	1,615,971	(716,605 -	6,368,048)	0
UKRI: UK Research and Innovation (UK)	93,002,486	19.1%	65	3.3%	706,245	(214,368 -	1,449,499)	0
National Natural Science Foundation of China (China)	50,376,238	10.4%	892	44.9%	34,764	(28,007 -	81,147)	0
European and Developing Countries Clinical Trial Partnership	42,393,026	8.7%	19	1.0%	1,793,524	(68,000 -	3,480,837)	0
CDC: Centers for Disease Control and Prevention	24,987,783	5.1%	7	0.4%	915,138	(716,540 -	1,802,981)	0
NIH: National Institutes for Health	21,198,893	4.4%	19	1.0%	415,262	(103,508 -	415,262)	0
São Paulo Research Foundation (Brazil)	12,855,231	2.6%	417	21.0%	9,263	(2,357 -	40,770)	0
Department of Biotechnology (India)	10,780,968	2.2%	38	1.9%	74,466	(112,790 -	112,790)	0
Wellcome Trust (UK)*	7,610,281	1.6%	24	1.2%	167,987	(68,883 -	467,499)	0
*Includes 4 grants jointly funded by DBT India Alliance								
**<u>Top 10 funding organizations or funder groups (by total number of grants awarded 2011-2020)</u>**								
National Natural Science Foundation of China (China)	50,376,238	10.4%	892	44.9%	0	(0 -	0)	0
São Paulo Research Foundation (Brazil)	12,855,231	2.6%	417	21.0%	9,263	(2,357 -	40,770)	0
National Council for Scientific and Technological Development (Brazil)	-	-	167	-	-	-		167
National Research Foundation (South Africa)	-	-	67	-	-	-		67
UKRI: UK Research and Innovation (UK)	93,002,486	19.1%	65	3.3%	706,245	(214,368 -	1,449,499)	0
Bill & Melinda Gates Foundation (USA)	117,116,089	24.1%	59	3.0%	355,314	(92,156 -	1,503,349)	0

Russian Foundation for Basic Research (Russia)	-	-	59	-	-	-		59
Department of Biotechnology (India)	10,780,968	2.2%	38	1.9%	74,466	(112,790 -	112,790)	0
European Union (European Commission/European Research Council) (EU)	97,461,227	20.0%	27	1.4%	1,615,971	(716,605 -	6,368,048)	0
Wellcome Trust (UK)**	7,610,281	1.6%	28	1.4%	167,987	(68,883 -	467,499)	0
World Health Organization	28,003	0.0%	22	1.1%	3,544	(2,748 -	11,253)	18
**Includes 4 grants jointly funded by DBT India Alliance								
**<u>Top 5 host countries of funding organizations or funder groups (by total active funding 2011-2020) (active funding *given*)</u>**								
United States	163,549,355	33.6%	95	4.8%	415,262	(98,761 -	1,503,349)	8
United Kingdom	101,624,421	20.9%	97	4.9%	371,336	(125,543 -	1,040,588)	0
Belgium†	97,759,758	20.1%	30	1.5%	1,471,885	(657,975 -	4,911,866)	2
China	56,319,040	11.6%	934	47.1%	35,841	(28,007 -	81,900)	2
Netherlands	42,393,026	8.7%	20	1.0%	1,793,524	(68,000 -	3,480,837)	1
Brazil	12,855,231	2.6%	584	29.4%	9,263	(2,357 -	40,770)	167
India	11,430,389	2.3%	54	2.7%	68,881	(32,185 -	109,611)	5
Canada	1,386,728	0.3%	8	0.4%	49,432	(20,394 -	320,557)	0
Switzerland††	457,052	0.1%	27	1.4%	11,798	(66,437 -	66,437)	20
Denmark	258,271	0.1%	4	0.2%	62,609	(50,754 -	78,381)	0
†USD $97461227 of funding from Belgium is from the EU (97%)<br>††USD $28003 of funding from Switzerland is from the WHO (6%)								

14. Figure S2. Bubble graph demonstrating relationship between neonatal mortality burden (mean annual neonatal deaths per 1000 live births) and total research investments received by LMIC institutions from 2011-2020.

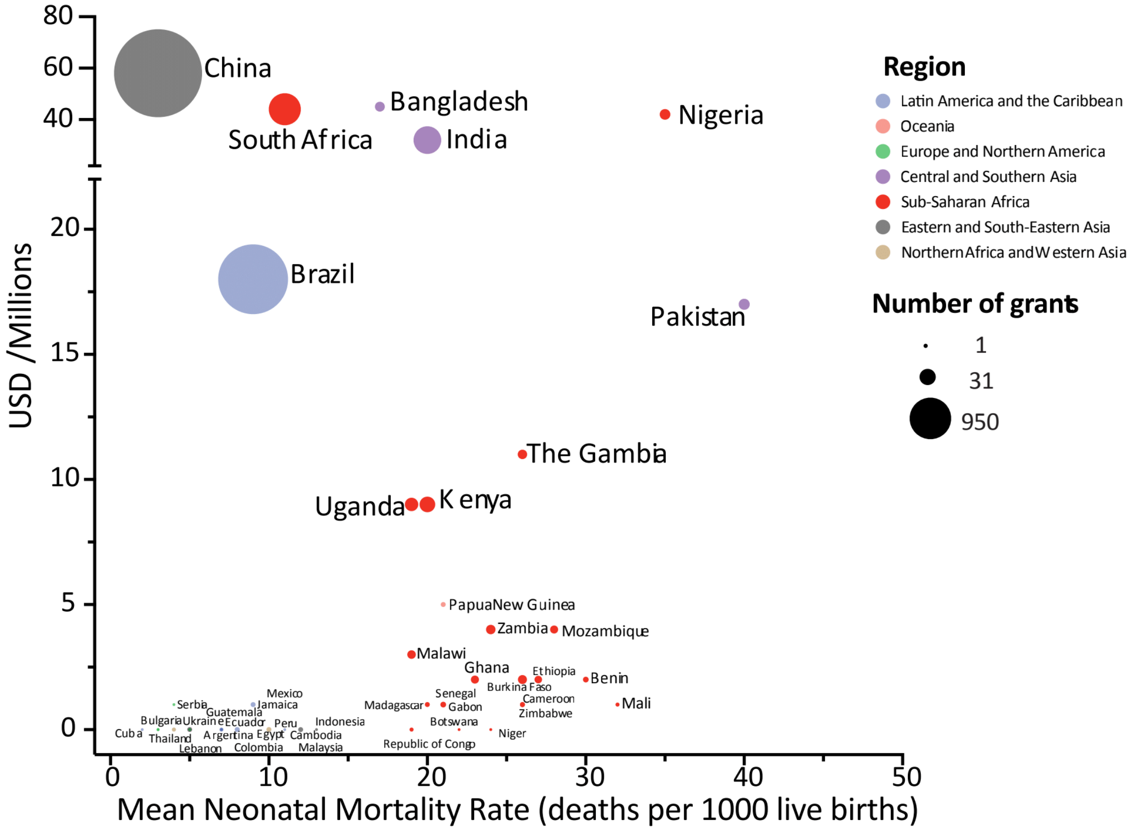

Figure S2: Mean neonatal mortality rate (2011-2020) (NMR) as defined by neonatal deaths per 1000 live births according to UN IGME data and total research investments received by LMIC institutions from 2011-2020 (funding amount and number of grants plotted are those specific to LMIC institutions and any funding apportioned to grant partners in HIC is not included here), with bubble size representative of number of grants received. NB: grant financial information was unavailable for 5.1% (345/6,790) of grants.

15. Figure S3. Newborn health and stillbirth research funding trends over time, and by thematic area

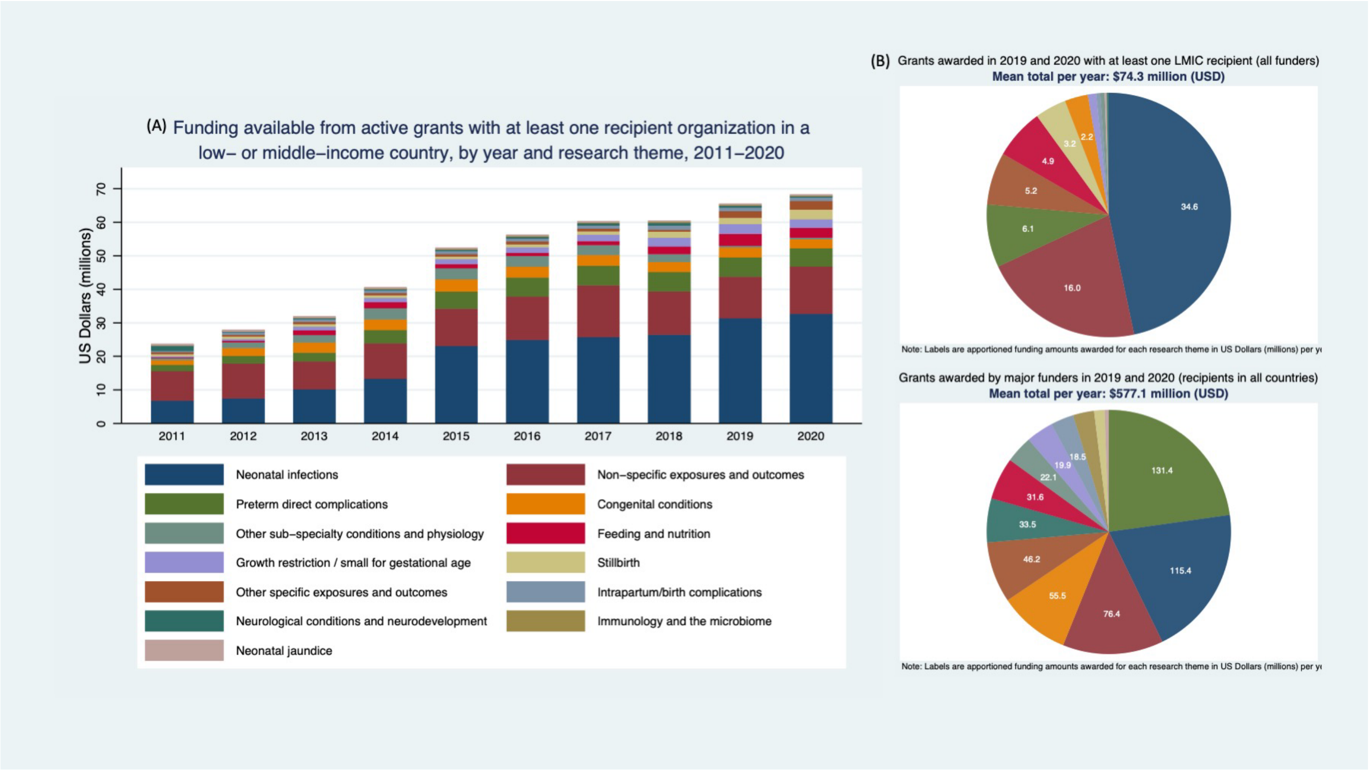

Figure S3: (A) Active funding takes into account all available funding per year, under the assumption that grants are evenly dispersed across their duration. Funding is adjusted for inflation according to the grant start date, to equate all amounts to the 2020 US dollar value. Funding awarded was assumed to be evenly distributed across categories, where individual grants covered multiple categories. (B) Pie charts show the total amounts newly committed to research in 2019 and 2020, comparing awards from all funders to research involving at least one LMIC recipient, and awards by major funders to recipients in all countries.

16. References

1. Oza S, Lawn JE, Hogan DR, Mathers C, Cousens SN. Neonatal cause-of-death estimates for the early and late neonatal periods for 194 countries: 20002013. *Bull World Health Organ* 2014; **93**: 19–28.
2. United Nations. SDG indicators - SDG indicators. https://unstats.un.org/sdgs/indicators/regional-groups/ (accessed 5.1.2023]
3. World Bank Group. 2020 World Bank Economy Status. https://data.worldbank.org/ (accessed 5.1.2023)
